# Two-component cyclase opsins of green algae are ATP-dependent and light-inhibited guanylyl cyclases

**DOI:** 10.1186/s12915-018-0613-5

**Published:** 2018-12-06

**Authors:** Yuehui Tian, Shiqiang Gao, Eva Laura von der Heyde, Armin Hallmann, Georg Nagel

**Affiliations:** 1 Botanik I, Julius-Maximilians-Universität Würzburg, Biozentrum, Julius-von-Sachs-Platz 2, 97082 Würzburg, Germany; 2 Department of Cellular and Developmental Biology of Plants, University of Bielefeld, Universitätsstr. 25, 33615 Bielefeld, Germany

**Keywords:** *Chlamydomonas reinhardtii*, *Volvox carteri*, Two-component system, Chlamyopsin, Optogenetics

## Abstract

**Background:**

The green algae *Chlamydomonas reinhardtii* and *Volvox carteri* are important models for studying light perception and response, expressing many different photoreceptors. More than 10 opsins were reported in *C. reinhardtii*, yet only two—the channelrhodopsins—were functionally characterized. Characterization of new opsins would help to understand the green algae photobiology and to develop new tools for optogenetics.

**Results:**

Here we report the characterization of a novel opsin family from these green algae: light-inhibited guanylyl cyclases regulated through a two-component-like phosphoryl transfer, called “two-component cyclase opsins” (2c-Cyclops). We prove the existence of such opsins in *C. reinhardtii* and *V. carteri* and show that they have cytosolic N- and C-termini, implying an eight-transmembrane helix structure. We also demonstrate that cGMP production is both light-inhibited and ATP-dependent. The cyclase activity of *Cr*2c-Cyclop1 is kept functional by the ongoing phosphorylation and phosphoryl transfer from the histidine kinase to the response regulator in the dark, proven by mutagenesis. Absorption of a photon inhibits the cyclase activity, most likely by inhibiting the phosphoryl transfer. Overexpression of *Vc*2c-Cyclop1 protein in *V. carteri* leads to significantly increased cGMP levels, demonstrating guanylyl cyclase activity of *Vc*2c-Cyclop1 in vivo. Live cell imaging of YFP-tagged *Vc*2c-Cyclop1 in *V. carteri* revealed a development-dependent, layer-like structure at the immediate periphery of the nucleus and intense spots in the cell periphery.

**Conclusions:**

*Cr*2c-Cyclop1 and *Vc*2c-Cyclop1 are light-inhibited and ATP-dependent guanylyl cyclases with an unusual eight-transmembrane helix structure of the type I opsin domain which we propose to classify as type Ib, in contrast to the 7 TM type Ia opsins. Overexpression of *Vc*2c-Cyclop1 protein in *V. carteri* led to a significant increase of cGMP, demonstrating enzyme functionality in the organism of origin. Fluorescent live cell imaging revealed that *Vc*2c-Cyclop1 is located in the periphery of the nucleus and in confined areas at the cell periphery.

**Electronic supplementary material:**

The online version of this article (10.1186/s12915-018-0613-5) contains supplementary material, which is available to authorized users.

## Background

The family of microbial rhodopsins expanded remarkably after the first demonstration of bacteriorhodopsin as a light-activated proton pump [1, 2] in the archaeon *Halobacterium halobium* [3] (later correctly identified as *H. salinarum*). Afterwards, the chloride-pumping halorhodopsin [4, 5], sensory rhodopsins [6], direct light-gated cation channels (channelrhodopsins) [7, 8], sodium ion pump rhodopsin [9], and anion channelrhodopsins [10] were found in archaea, bacteria, and eukaryotes.

The first microbial rhodopsin with proven enzyme activity is *Be*Cyclop from the fungus *Blastocladiella emersonii*, first described as a possibly light-activated guanylyl cyclase (*Be*GC1) by studies of the protein in the fungus [11]. After heterologous expression and thorough characterization as light-activated guanylyl cyclase, it was then named cyclase opsin (Cyclop) [12], RhGC (rhodopsin-guanylyl cyclase) [13], or RhoGC (rhodopsin-guanylyl cyclase) [14, 15]. *Be*Cyclop is the first rhodopsin with proven cytosolic N- and C-termini and most likely eight transmembrane helices (TMs), as first proposed and demonstrated by us [12] and later confirmed by Trieu et al. with a different method [15].

Another new enzymatic microbial rhodopsin is the rhodopsin phosphodiesterase, abbreviated as RhPDE [16] or RhoPDE [17, 18]. We demonstrated that RhoPDE is light-regulated via a light-sensitive Michaelis-Menten constant [18]. Interestingly, we and Lamarche et al. also find that RhoPDE shows cytosolic N- and C-termini [17, 18] with an additional N-terminal transmembrane helix. We now propose to classify the microbial or type I rhodopsins in two subtypes: type Ia with 7 TM helices and an extracellular N-terminus and type Ib with 8 TM helices and a cytosolic N-terminus.

The genome of the green alga *C. reinhardtii* encodes many photoreceptors and several opsins, which were provisionally named chlamyopsin-1 to 7 (Cop1 to Cop7) [19] and recently extended to Cop1 to Cop12 [20]. But until now, functional heterologous expression was demonstrated only for Cop3 and 4 [7, 8] (see below). Whereas Cop1 and Cop2 were cloned as opsins due to their retinal binding capacity, they show no similarity to the later identified type I opsins, also termed chlamyopsins (Cop3–12). Cop1/2 were originally proposed to be the long missing phototaxis sensors, closely connected to a light-gated passive conductance [21], but at present, their function is completely unclear. RNAi experiments clearly showed that they are not involved in phototaxis [22], and to call them opsins is disputable, even if a sequence similarity to invertebrate opsins was claimed in the first publication [21]. However, considering the lacking similarity to the other chlamyopsins with a microbial opsin domain and the lacking membrane association of Cop1 and Cop2 (see the “Results” section), we propose that they should not be called “opsins” anymore. Cop-3 and 4 were then the first real opsins, found in a *C. reinhardtii* EST database due to their similarity to bacteriorhodopsin [7, 23, 24]. After heterologous expression in oocytes of *Xenopus laevis*, they were shown to code for directly light-gated cation channels and were therefore named channelopsin-1 and 2 (chop1/2) [7, 8].

Previous to the discoveries of Cyclop and RhoPDE, a third group of rhodopsins with enzyme function was predicted to exist in *C. reinhardtii* due to EST and genome data [19]: Cop5, Cop6, and Cop7. These opsins were predicted to be very large, with an N-terminal opsin domain, followed by a histidine kinase, a response regulator, and a cyclase domain. Such structures consisting of a receptor, followed by a histidine kinase, a response regulator, and an output domain—often as single domain, sometimes fused together to bigger proteins—are known from bacteria and plants as two-component systems (TCS or 2c). In analogy, these predicted new opsins in *Chlamydomonas* with fused 2c domains suggested a signaling cascade with light induction, phosphoryl transfer, and light-regulated cyclase activity. We now call the proteins (previously predicted as Cop6 in *Chlamydomonas reinhardtii* and Vop6 in *Volvox carteri*) after functional characterization (see below) two-component cyclase opsins or 2c-Cyclops.

Although predicted to exist in green algae for more than 12 years, nobody was able to demonstrate the cyclase activity until now and light regulation of the proposed cyclase was also only a speculation. So far, only the opsin part of one of these 2c-Cyclops from *C. reinhardtii* (provisionally named “chlamyopsin-5” or “Cop5”) was heterologously expressed (named “histidine kinase rhodopsin” or “HKR”), and a very slow photocycle with “switch-like” absorption between UV and visible light was shown [25–28].

Here we report the cloning and characterization of two complete 2c-Cyclop proteins, *Cr*2c-Cyclop1 from *C. reinhardtii* and *Vc*2c-Cyclop1 from *V. carteri.* We were able to measure guanylyl cyclase activity in the light and in the dark whereby, unexpectedly, the 2c-Cyclop proteins turned out to be light-inhibited guanylyl cyclases. Interestingly, the 2c-Cyclop action spectra peak between the absorption peaks of photosynthesis and the cGMP production of 2c-Cyclop is very sensitive to light and needs ATP (in addition to GTP) to support GC activity.

Guanylyl cyclase activity of *Vc*2c-Cyclop1 was verified in vivo by overexpression of *Vc*2c-Cyclop1 protein in *V. carteri*, which leads to significantly increased cGMP levels. In addition, the cGMP concentration roughly correlates with the *Vc*2c-Cyclop1 mRNA expression rate. Live cell imaging of YFP-tagged *Vc*2c-Cyclop1 revealed a development-dependent, layer-like structure at the immediate periphery of the nucleus and intense spots further away.

Furthermore, the 2c-Cyclops also have 8 TMs like RhoPDE and the family of fungal Cyclops and therefore belong to the new class of type Ib opsins. These three type Ib rhodopsin families with 8 TMs, light-activated guanylyl cyclase (Cyclop), light-inhibited guanylyl cyclase (2c-Cyclop), and light-activated PDE (RhoPDE) activity seem to be precursors to the more sophisticated light regulation of cGMP concentration by type II rhodopsins in the mammalian vision system. The newly discovered type Ib rhodopsins offer versatile possibilities to regulate cGMP concentration by illumination.

## Results

### The “chlamyopsins” Cop1 and Cop2 are no membrane proteins

The *C. reinhardtii* genome contains several opsins which were provisionally named chlamyopsins [19, 20]. Due to unclear function of Cop1 and Cop2, we synthesized their DNA, based on database-derived amino acid sequence (Additional file 1: Figure S1A), for expression in *Xenopus* oocytes and further study. After 3 days of expression of YFP-tagged Cop1 and Cop2, both proteins can be found exclusively in the soluble fraction while the proven membrane protein channelopsin-2 (Chop2-YFP) is only found in the membrane fraction (Additional file 1: Figure S1B and C). As reported previously [29], all-trans-retinal (ATR) addition to the medium strongly enhanced the expression, i.e., degradation resistance, of Chop2-YFP but did not influence the expression of YFP-Cop1 and YFP-Cop2.

The lack of membrane insertion of Cop1/2 is immediately understandable when considering their high content of lysines: it is more than 15%, whereas other opsins typically show a lysine content of only 2–3%. On the other hand, the high lysine content might explain their excellent retinal binding capacity, which, at the time, enabled the identification of Cop1 as a [^3^H] retinal-labeled protein, a supposed opsin, and cloning of its cDNA [21]. Further investigation will have to find out if cop1/2 are not only no opsins but also no photoreceptors, as current experimental data suggest.

### Genes and mRNAs of *Cr*2c-Cyclop1 and *Vc*2c-Cyclop1

**Fig. 1:**
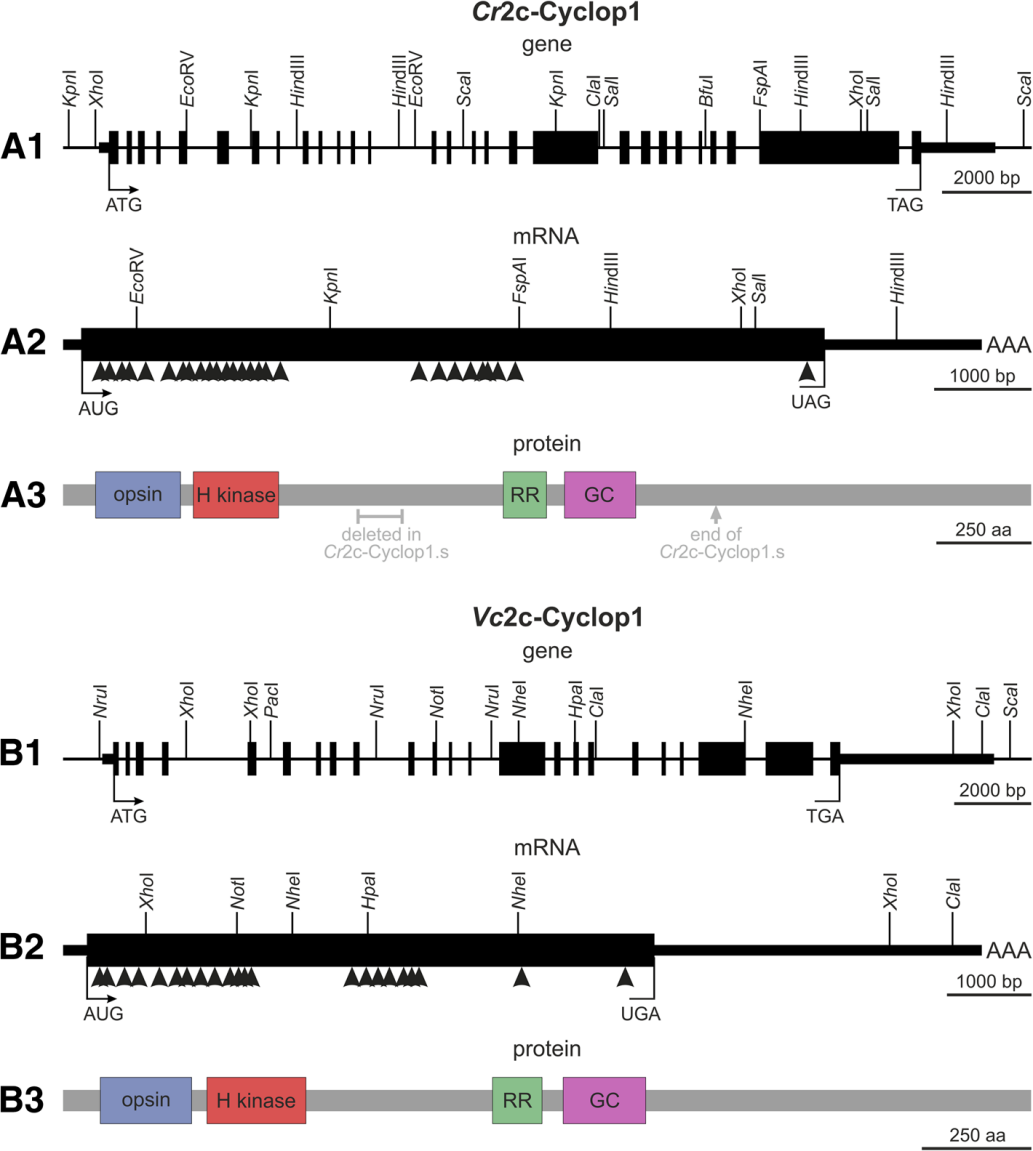
Gene structures, mRNA structures, and protein domain compositions of *Cr*2c-Cyclop1 and *Vc*2c-Cyclop1. **A1**, **B1**. The genetic maps of *Cr*2c-Cyclop1 and *Vc*2c-Cyclop1 genes show all exon and intron segments and a number of restriction enzyme cleavage sites. ATG, the translation start site; TAG or TGA, the translation stop site; the filled boxes represent exons; thick horizontal bars indicate untranslated regions; and the thinner horizontal bars represent upstream and downstream sequences. **A2**, **B2**. Arrowheads indicate the position of the numerous introns within the genetic maps of the *Cr*2c-Cyclop1 and *Vc*2c-Cyclop1 mRNAs. The specified restriction enzyme cleavage sites facilitate orientation. AUG, the translation start site; UAG or UGA, the translation stop site; filled black box, the open reading frame; thick horizontal bar, the 5′- and 3′-UTRs; AAA, the poly A tail. **A3**, **B3**. *Cr*2c-Cyclop1 and *Vc*2c-Cyclop1 protein domain compositions. Opsin, the rhodopsin domain; H kinase, the histidine kinase domain; RR, the response regulator domain; GC, the guanylyl cyclase domain. Gray text in A3 shows where *Cr*2c-Cyclop has been shortened to produce *Cr*2c-Cyclop1

Searching the genome databases of *C. reinhardtii* and *V. carteri* on Phytozome, the plant comparative genomics portal of the Department of Energy’s Joint Genome Institute (DOE JGI), revealed sequences that contain both protein domains related to guanylyl cyclase opsins and additional domains: *Cr*2c-Cyclop1 and *Vc*2c-Cyclop1. Phytozome gene annotations, genomic sequences, and cDNA sequences of generated RT-PCR products were used to obtain the complete coding sequences of both 2c-Cyclop genes. Because of the enormous sizes, the required full-length cDNAs were finally amplified in overlapping parts by RT-PCR, using total RNA from wild-type algae. The determined lengths of the mRNAs were 9464 bp for *Cr*2c-Cyclop1 and 10,885 bp for *Vc*2c-Cyclop1 with open reading frames of 7650 bp for *Cr*2c-Cyclop1 and 6699 bp for *Vc*2c-Cyclop1 (Fig. 1A2, B2). The comparison between mRNA and genomic sequences shows that the *Cr*2c-Cyclop1 and *Vc*2c-Cyclop1 genes contain 27 and 22 introns, respectively (Fig. 1A1, B1). The genomic sequence that codes for *Cr*2c-Cyclop1 mRNA covers 19.5 kb; the 28 exons are between 50 and 3005 bp in length, whereby 25 exons are smaller than 250 bp. The genomic sequence that codes for *Vc*2c-Cyclop1 mRNA covers 22.8 kb; the 23 exons are between 73 and 4223 bp in length, whereby 19 exons are smaller than 250 bp.

### Proteins of *Cr*2c-Cyclop1 and *Vc*2c-Cyclop1

The open reading frames of *Cr*2c-Cyclop1 and *Vc*2c-Cyclop1 mRNAs encode proteins of 2549 and 2210 aa with expected molecular masses of 256.55 and 232.42 kDa, respectively. Amino acid residues 66 to 303 of *Cr*2c-Cyclop1 define a microbial-type opsin domain with eight, instead of the “classical” seven, putative transmembrane helices (see below) (Fig. 1A3). The same condition with eight putative membrane spanning α-helices applies to amino acid residues 49 to 287 of *Vc*2c-Cyclop1 (Fig. 1B3). The 2c-Cyclop proteins have a histidine kinase domain directly behind the opsin domain, followed by an intermediate section with approximately 500 aa of unknown function. Next comes a response regulator domain, directly followed by a guanylyl cyclase domain (Fig. 1A3, B3). For the last, C-terminal section with 1160 aa in *Cr*2c-Cyclop1 and 885 aa in *Vc*2c-Cyclop1, the function is again unknown.

When comparing one with another, the 2c-Cyclop proteins show 69% identity and 78% similarity in a 737-aa fragment that contains the opsin and histidine kinase domains (Additional file 2: Figure S2). Likewise, in a 641-aa fragment that contains the response regulator and guanylyl cyclase domains, both proteins show 67% identity and 73% similarity. Outside the mentioned domains, the similarities between both proteins are quite low, except for a short, 75-aa fragment close to the C-terminus, in which they show 67% identity and 81% similarity.

For heterologous expression in oocytes, different variants of shortened *Cr*2c-Cyclop1 were constructed as the cloned DNA construct was shorter than the predicted ORF from genomic data (see below). The finally used variant, *Cr*2c-Cyclop1.s, corresponds to the cDNA cloned by us from *C. reinhardtii* RNA and is shown in (Fig. 1A3). For homologous expression in *V. carteri*, a full-length clone of *Vc*2c-Cyclop1 was produced, covering the complete coding sequence and the first two introns contained therein.

### 2c-Cyclop proteins are membrane-embedded with cytosolic N- and C-termini and 8 predicted TM helices

TMHMM analysis of the 2c-Cyclop opsin part and alignment with other microbial opsins suggest that 2c-Cyclop has 8 transmembrane (TM) helices (Additional file 3: Figure S3), which is similar to the other two enzyme rhodopsin families, Cyclop and RhoPDE.

**Fig. 2:**
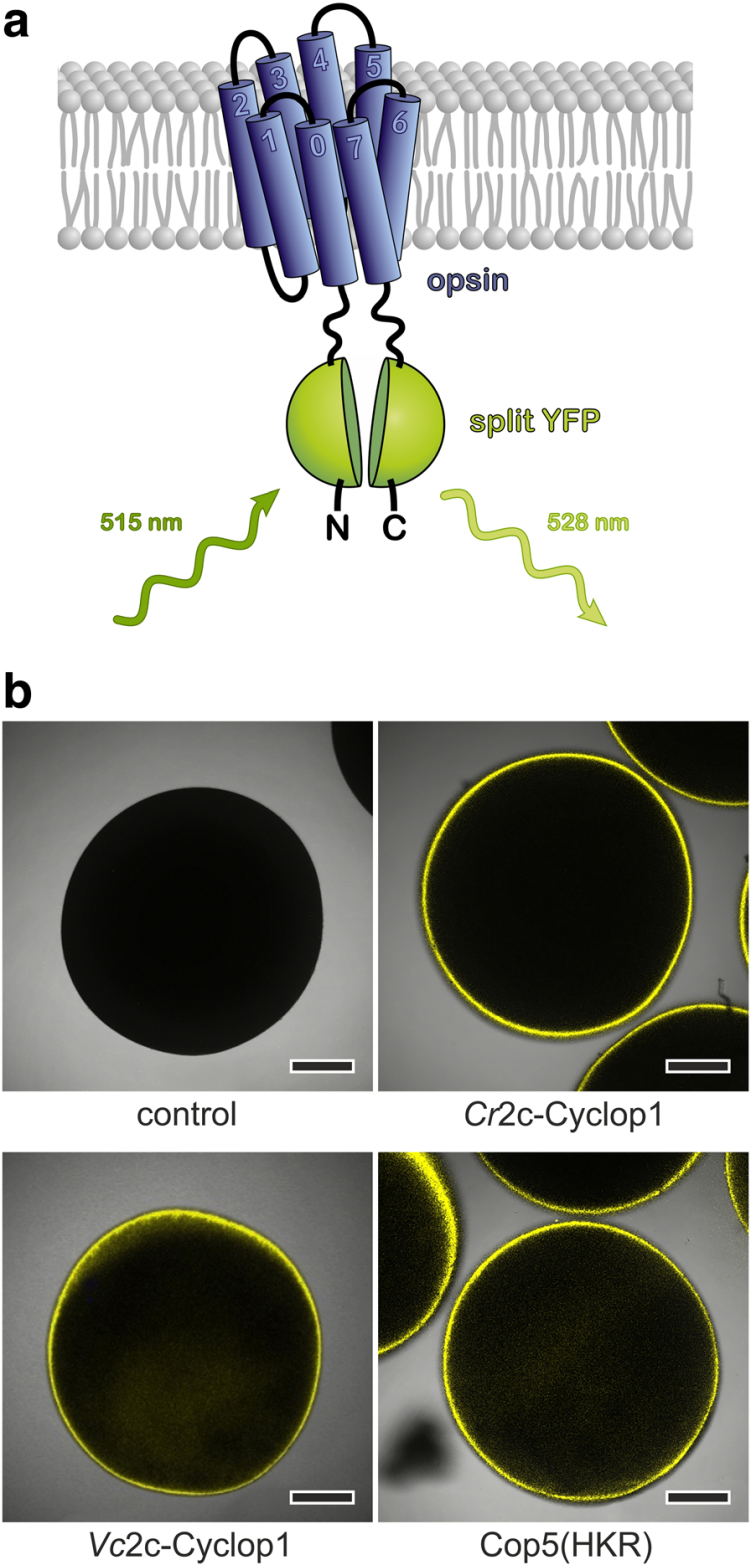
2c-Cyclops possess cytosolic N-termini and a likely 8 transmembrane helices topology. **a** Schematic model of BiFC (bimolecular fluorescence complementation) experiments. The opsin domain was N- and C-terminally fused to the two parts of split YFP (YFP_C_ = aa155–238 of YFP, YFP_N_ = aa1–154 of YFP). **b** Fluorescence pictures show the following: control oocyte (control), oocytes expressing YFP_C_::*Cr*2c-Cyclop1/opsin::YFP_N_ (*Cr*2c-Cyclop1), YFP_C_::*Vc*2c-Cyclop1/opsin::YFP_N_ (*Vc*2c-Cyclop1), and YFP_C_::Cop5/opsin::YFP_N_ (Cop5(HKR)) constructs. The fusion sequence of YFP_C_ and YFP_N_ was designed according to the YFP structure to facilitate the fluorescence complementation. The fluorescence images were taken by a confocal microscope 3 dpi (days post injection) with 30 ng cRNA injection into *Xenopus* oocytes. Scale bars 250 μm

To confirm the cytosolic localization of the 2c-Cyclops N-termini experimentally, bimolecular fluorescence complementation (BiFC) constructs with split YFP were made using the opsin part of the 2c-Cyclops (Fig. 2a) and the published BiFC vector [12, 18]. Amino acid residues 1 to 340 of *Cr*2c-Cyclop1, 2 to 302 of *Vc*2c-Cyclop1, and 2 to 320 of Cop5 (or “HisKR”) were used to generate the BiFC fusion constructs with YFP fragments. YFP fluorescence could be clearly seen after expressing these constructs in *Xenopus* oocytes, which indicated that the N- and C-termini locate on the same side of the membrane (Fig. 2b). Taken together, based on the TMHMM prediction, opsin part alignment, BIFC experiment results, and cytosolic cGMP production, we can conclude that *Cr*2c-Cyclop1, *Vc*2c-Cyclop1, and Cop5 have 8 TM helices and are therefore type Ib opsins.

### 2c-Cyclops are light-inhibited guanylyl cyclases

Alignment of the predicted mononucleotide cyclase part of 2c-Cyclop with CYG12 [30], bPAC [31], and Cya2 [30, 32] showed high identity in positions important for guanylyl cyclase function (Additional file 4: Figure S4). This suggests 2c-Cyclop to function as a guanylyl cyclase.

**Fig. 3:**
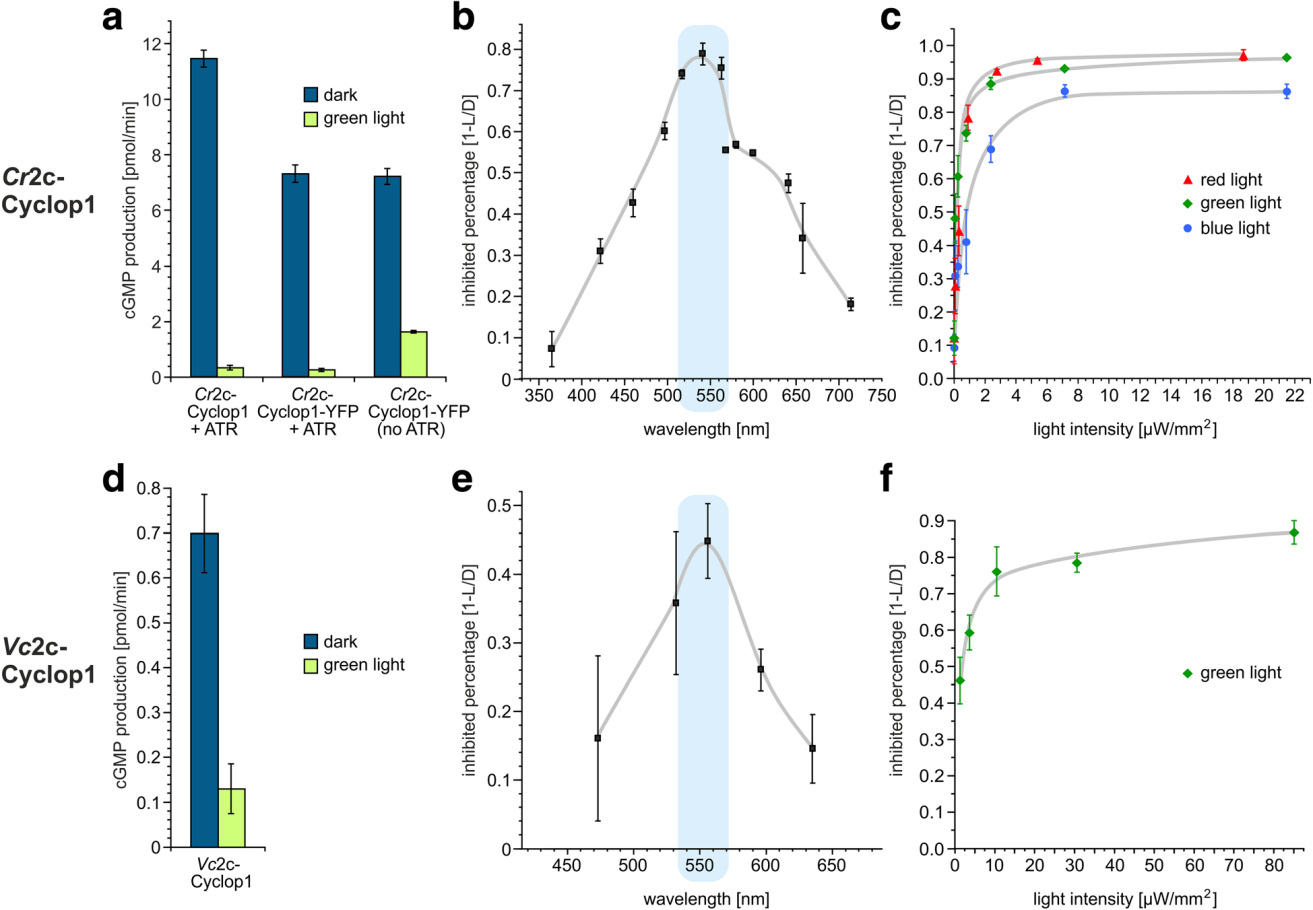
*Cr*2c-Cyclop1 and *Vc*2c-Cyclop1 are light-inhibited guanylyl cyclase opsins. **a** Light (532 nm, ~ 20 μW/mm^2^) and dark activities of *Cr*2c-Cyclop1 + 1 μM ATR, *Cr*2c-Cyclop1-YFP + 1 μM ATR, and *Cr*2c-Cyclop1-YFP without ATR. The cGMP production ability was normalized to one oocyte membrane. *n* = 3, error bars = SD. **b** Action spectrum of *Cr*2c-Cyclop1. *n* = 3, error bars = SD. **c** Light sensitivity of *Cr*2c-Cyclop1 to three different wavelengths of light. Different intensities of blue light (473 nm), green light (532 nm), and orange light (596 nm) were applied. *n* = 3, error bars = SD. **d** Light (532 nm, ~ 20 μW/mm^2^) and dark activities of *Vc*2c-Cyclop1. One micromolar of ATR was added. The cGMP production ability was normalized to one oocyte membrane. *n* = 4, error bars = SD. **e** Action spectrum of *Vc*2c-Cyclop1. *n* = 4, error bars = SD. **f** Light sensitivity of *Vc*2c-Cyclop1 to 556 nm light. *n* = 4, error bars = SD. For **b**, **c**, **e**, and **f**, inhibition percentage was calculated by (dark activity − light activity)/dark activity

In vitro reactions were designed for characterizing 2c-Cyclop with *Xenopus* oocyte membranes expressing 2c-Cyclop. As shown in Fig. 3a, the *Cr*2c-Cyclop1 has high guanylyl cyclase activity in the dark and, moreover, the guanylyl cyclase activity was inhibited by illumination with green light (532 nm, 20 μW/mm^2^). No cAMP production of *Cr*2c-Cyclop1 was detectable under any condition. This suggested *Cr*2c-Cyclop1 to be a light-inhibited guanylyl cyclase.

The sequence of the amplified *Cr*2c-Cyclop1 cDNA was different in the middle part and C-terminus from the prediction in the JGI database (Fig. 1A1–A3, Additional file 5: Figure S5A). Before we continued with a more detailed study, we tested *Cr*2c-Cyclop1 with four different lengths to investigate how the different middle parts and C-termini would influence the *Cr*2c-Cyclop1 function. Results showed that all four constructs have light-inhibited guanylyl cyclase activity, but the one we got from cDNA showed the highest activity and also the best D/L (dark activity to light activity) ratio (Additional file 5: Figure S5B and S5C). Therefore, in further work, we used this construct with a shortened C-terminal and a shortened middle sequence, compared to the full-length sequence, as derived from genomic data (*Cr*2c-Cyclop1.s vs. *Cr*2c-Cyclop1.fl in Fig. 1A1–A3 and Additional file 5: Figure S5A). The following data on *Cr*2c-Cyclop1 always refer to this *Cr*2c-Cyclop1.s sequence. The reaction condition was then optimized since *Cr*2c-Cyclop1 showed higher D/L ratio with 100 mM NaCl (Additional file 5: Figure S5D). *Vc*2c-Cyclop1 also was amplified from cDNA and used in full length for all studies (Fig. 1B1–B3).

### Light regulation of 2c-Cyclop

In the dark, *Cr*2c-Cyclop1-expressing membranes from one oocyte could produce 11 ± 0.3 pmol cGMP/min in the reaction mix. With 20 μW/mm^2^ 532 nm light illumination, the activity was reduced to 0.3 ± 0.09 pmol cGMP/min (Fig. 3a). The D/L ratio is therefore ~ 35. A C-terminal YFP tag slightly reduces the guanylyl cyclase activity to ~ 65%, possibly due to a reduced protein production because the D/L ratio remained unchanged (Fig. 3a). An in vitro assay with *Vc*2c-Cyclop1, expressed in *Xenopus* oocytes, showed that *Vc*2c-Cyclop1 also exhibited light-inhibited guanylyl cyclase activity (Fig. 3d). The D/L ratio of *Vc*2c-Cyclop1 was ~ 5.

Oocytes of *Xenopus laevis* contain endogenous all-trans-retinal (ATR), but for many heterologously expressed opsins, this ATR is not sufficient for optimal reconstitution to the corresponding rhodopsin. Therefore, ATR has to be added to the medium [29] during the expression time (usually 1 μM ATR for 3 days of expression). When no ATR was added to the medium, the membranes from *Cr*2c-Cyclop1-expressing oocytes have higher guanylyl cyclase activity under illumination than with added ATR during expression, while the dark activity is similar (Fig. 3a). Therefore, the D/L ratio decreases to ~ 4.5 without added ATR. The less tight inhibition by light without added ATR suggested that *Cr*2c-Cyclop1 without bound ATR is a functional but not light-sensitive guanylyl cyclase. We conclude that ATR binding to the opsin domain is important for *Cr*2c-Cyclop1 to be strictly inhibited by light.

To further test the wavelength dependence of *Cr*2c-Cyclop1, light with different wavelengths and similar photon density was used to measure the activity. An action spectrum was then obtained by calibration to equal photon density. As shown in Fig. 3b, light at ~ 540 nm shows the strongest inhibition effect for *Cr*2c-Cyclop1. Accordingly, the inhibition is less effective with blue, UV, and red light.

*Cr*2c-Cyclop1 is very sensitive to light, compared to other nucleotidyl cyclase photoreceptors such as *Be*Cyclop, mPAC, and bPAC. The half-maximal inhibition (*K*_0.5_) light intensity was determined to be ~ 0.2 μW/mm^2^ with both 532 nm and 596 nm light (Fig. 3c). The *K*_0.5_ increases to ~ 0.5 μW/mm^2^ with 473 nm light, which is less effective to inhibit *Cr*2c-Cyclop1. In comparison, the half-maximal activation (*K*_0.5_) was observed at 55 μW/mm^2^, 4 μW/mm^2^, and 6 μW/mm^2^ for *Be*Cyclop [12], bPAC [31], and mPAC [33], respectively, with light wavelengths near the peak of their action spectra. To our knowledge, *Cr*2c-Cyclop1 is so far the most sensitive nucleotide cyclase photoreceptor.

Tests with different wavelengths showed that the *Vc*2c-Cyclop1 action spectrum peaks at ~ 550 nm, which is close to that of *Cr*2c-Cyclop1 (Fig. 3e). *Vc*2c-Cyclop1 is less sensitive to light than *Cr*2c-Cyclop1 with a half-maximal inhibition of ~ 1.3 μW/mm^2^ to 556 nm light, which is close to its action spectrum peak (Fig. 3f).

Considering the better performance of *Cr*2c-Cyclop1, expressed in *Xenopus* oocytes, i.e., a higher D/L ratio, which facilitates data analysis, we focused on *Cr*2c-Cyclop1 for further studies on the functional mechanism of 2c-Cyclop1.

### Photocycle turnover time of functional *Cr*2c-Cyclop

Previously, it was suggested that the 2c-Cyclop-related protein Cop5 shows a switch-like photocycle, which needs light activation of a photocycle intermediate to return to the “ground state” [27]. However, no functional output of Cop5 was measured as expression of the full-length protein was not successful. The analysis depended on absorption changes of the isolated rhodopsin domain, which was heterologously expressed. We cloned the full-length cDNA of Cop5 from *Chlamydomonas*, which was identical to the corresponding JGI database sequence (Cre02.g074150.t2.1). The full-length Cop5 could be expressed in *Xenopus* oocytes with a C-terminal YFP tag. But no GC or AC activity could be detected for Cop5, neither in the dark nor in the light. In fact, the Cop5 cyclase domain showed very poor similarity to other functional GC or AC in nearly all the key residues (Additional file 4: Figure S4).

**Fig. 4:**
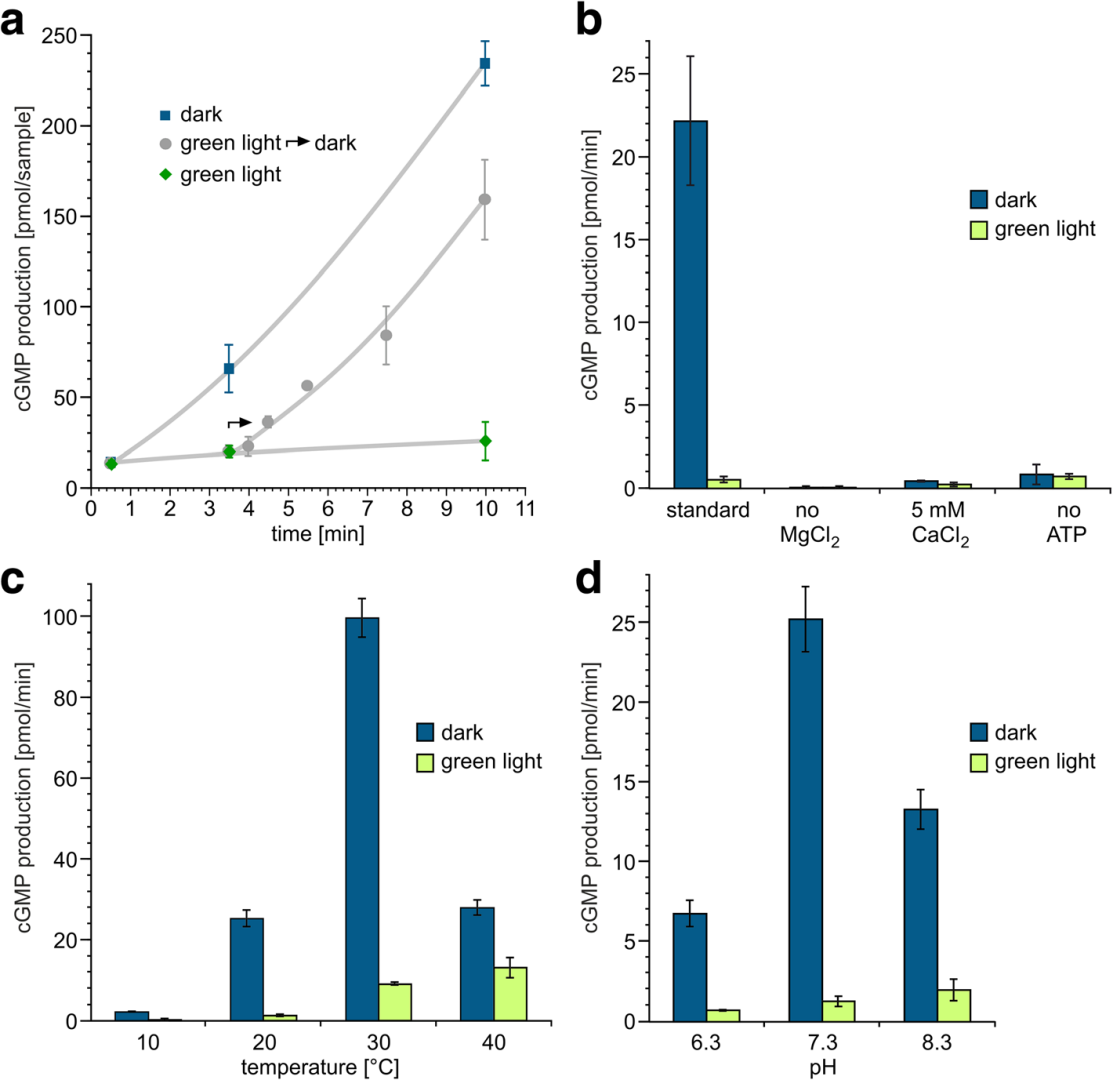
Characterization of *Cr*2c-Cyclop1 activity under different conditions. **a** Dynamic activity of *Cr*2c-Cyclop1. All three samples were under initial dark stage for 30 s and then put to constant dark (blue square), constant green light illumination (532 nm, ~ 20 μW/mm^2^, green rhombus), and 3-min green light followed by constant dark (gray dot). Samples were collected and measured at different time points indicated in the figure. *n* = 3, error bars = SD. **b** *Cr*2c-Cyclop1 activity influenced by ATP, Mg^2+^, and Ca^2+^. Mg^2+^ were depleted (no MgCl_2_), replaced by Ca^2+^ (5 mM CaCl_2_) or ATP depleted (no ATP) from the standard reaction buffer. *n* = 4, error bars = SD. **c** *Cr*2c-Cyclop1 activity at different temperatures (10, 20, 30, and 40 °C). *n* = 3, error bars = SD. **d** *Cr*2c-Cyclop1 activity at different pHs (6.3, 7.3, 8.3). *n* = 3, error bars = SD. Illumination conditions were same for **a**–**d**

For *Cr*2c-Cyclop1, we set out to estimate photocycle turnover time by measuring the time dependence of activity switching when changing from light to dark. By measuring the cGMP production at different time points and starting the reaction in light, then changing to dark condition at a certain time point, we could observe that the cGMP production changes back to the same rate as under constant dark condition within ~ 30 s (Fig. 4a). Therefore, we can conclude that the photocycle of the rhodopsin is not “switch-like” but recovers to the dark state with a time constant of ≤ 30 s at 20 °C.

### Guanylyl cyclase activity of *Cr*2c-Cyclop1 under different reaction conditions

Nucleotidyl cyclase activity and phosphoryl transfer are normally modulated by divalent cations, such as Mg^2+^ and Ca^2+^. Our standard in vitro assay was performed with 5 mM Mg^2+^. When Mg^2+^ was removed (with EDTA), the cyclase activity under both dark and light condition becomes non-detectable (Fig. 4b). When Mg^2+^ was replaced by Ca^2+^, the dark activity decreased to ~ 1/50 of the +Mg^2+^ condition, while the light activity remained nearly unchanged (Fig. 4b). However, it is currently unclear if Ca^2+^ impacts the cyclase activity or the phosphoryl transfer from histidine to aspartic acid or both (see below).

The pH and temperature are also important parameters for enzymatic function; both influenced the activity and D/L ratio. A temperature increase from 10 to 30 °C increased the dark activity (Fig. 4c), whereas a further increase to 40 °C impaired the dark activity. But the best D/L ratio, which indicates the tightness of the light regulation, was obtained in ~ 20 °C. The highest D/L ratio and dark activity was obtained at pH 7.3 (Fig. 4d). When pH was changed to 6.3 or 8.3, the dark activity decreased, and the D/L ratio dropped to < 10.

### Light-regulating mechanism inside *Cr*2c-Cyclop1

**Fig. 5:**
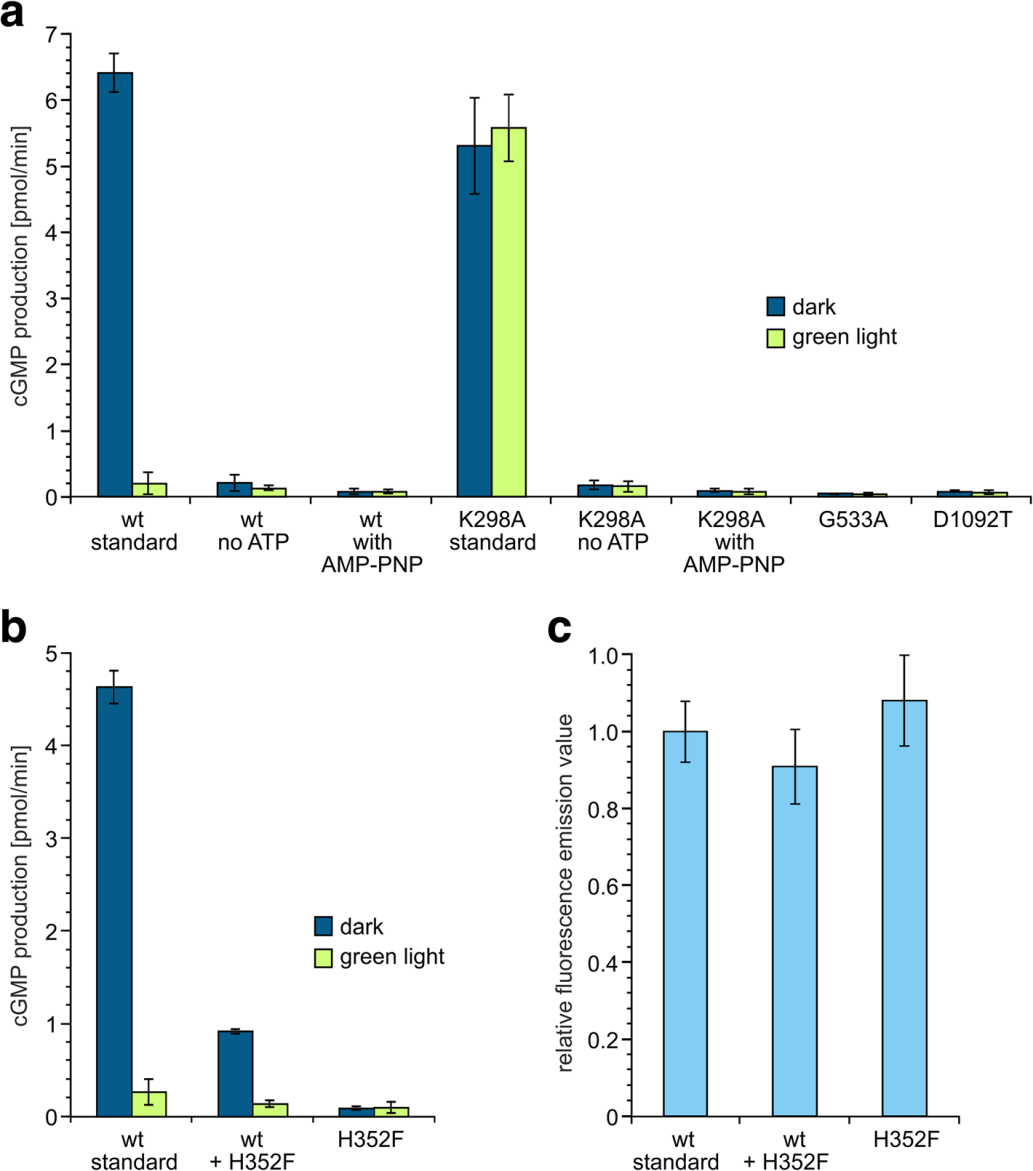
Mutation analysis of *Cr*2c-Cyclop1. **a** All mutations were made based on *Cr*2c-Cyclop1-YFP construct. No ATP, ATP depleted from the standard reaction buffer. With AMP-PNP, 0.25 mM AMP-PNP was added to replace ATP. Illumination condition, 532 nm, ~ 20 μW/mm^2^. Activities of different constructs were adjusted to the same protein amount based on the fluorescence emission value, *n* = 4, error bars = SD. wt, *Cr*2c-Cyclop1-YFP wild-type. **b** Co-expression of *Cr*2c-Cyclop1-YFP and H352F mutant. Total protein amounts were controlled to be the same based on the fluorescence emission value. Illumination condition, 532 nm, ~ 20 μW/mm^2^. *n* = 3, error bars = SD. **c** The relative fluorescence emission values of different constructs were determined to ensure similar total protein amount. *n* = 3, error bars = SD

Whereas for the light-sensitive guanylyl cyclase *Be*Cyclop it has to be assumed that a conformational change of the opsin is transmitted via the coiled coil domain to directly activate the cyclase, a phosphoryl transfer can be presumed as signal in the case of 2c-Cyclop. A typical two-component system will need ATP to provide a high-energy phosphoryl group for phosphorylation of a conserved histidine and will transfer it upon stimulation to a conserved aspartic acid. Not surprisingly, when ATP was omitted from the in vitro assay, light regulation of *Cr*2c-Cyclop1-mediated cGMP production was not observed (Fig. 5a). However, in the light and the dark, *Cr*2c-Cyclop1 activity in ATP absence was dramatically reduced and even slightly lower than *Cr*2c-Cyclop1 activity with 0.25 mM ATP under illumination (20 μW/mm^2^ 532 nm). Therefore, we conclude that ATP is needed in the dark to activate the guanylyl cyclase activity. This means that the phosphoryl transfer, i.e., the phosphorylated aspartic acid, should activate the guanylyl cyclase activity and that the phosphoryl transfer is inhibited by light. Further mutation analysis was performed to prove this hypothesis, as shown below.

Alignment with other microbial opsins, histidine kinases, and response regulators, which constitute the two-component system, suggested several amino acids that might play important roles for regulation of the *Cr*2c-Cyclop1 activity. As shown in Additional file 3: Figure S3, the lysine at position 298 (K298) should be important for the retinal binding of the opsin. The histidine at position 352 (H352), located in the core of the H box, should be important for the kinase activity to transfer the phosphoryl group while the threonine at position 356 (T356) was supposed to function mostly as a phosphatase [34] (Additional file 6: Figure S6). The glycine at position 533 (G533) is suggested to be located in the G box of the histidine kinase which might be important for the binding and hydrolysis of ATP to provide the phosphoryl group (Additional file 6: Figure S6). The aspartic acid at position 1092 (D1092) is predicted to be the key residue in the response regulator to receive the phosphoryl and regulate the downstream activity (Additional file 6: Figure S6). A mutation analysis at these positions was conducted to substantiate the above hypothesis.

Mutation of K298 to alanine (K298A) destroyed the light inhibition of *Cr*2c-Cyclop1. Both the dark and light activities are close to the dark activity of the wild type (wt) (Fig. 5a). Depletion of ATP during the reaction caused a decrease of K298A activity to the light-inhibited level of wt. Addition of the non-hydrolyzable ATP analog AMP-PNP cannot restore the activity. The K298A mutation needs ATP to reach the activity of wt in the dark. This result proved that the phosphoryl transfer in the dark is necessary to keep the guanylyl cyclase working.

The H352F mutation, which should break the phosphoryl transfer, led to a low activity in the dark, similar to wt during illumination (Fig. 5b). A further G533A mutant, which should not be able to bind and hydrolyze ATP, also led to inhibited activity (Fig. 5a). Light regulation was compromised in both mutants. The inhibited activity, caused by blocking the ATP hydrolysis (providing phosphoryl) or the phosphoryl transfer, indicated that the guanylyl cyclase activity requires the phosphorylation of the aspartic acid in the response regulator domain.

Mutating D1092 was predicted to hamper the phosphorylation of the response regulator. The D1092T mutant had activity (in the dark and in the light) similar to light-inhibited wt activity (Fig. 5a). This suggested that the phosphorylation of D1092 is necessary for the dark-enhanced guanylyl cyclase activity.

Nucleotide cyclases of type III need to form a dimer to function. To test the dimerization of *Cr*2c-Cyclop1, we expressed the wt and H352F mutant together to check the guanylyl cyclase activity. The protein amount was monitored by the fluorescence emission of a C-terminally fused YFP (Fig. 5c) to ensure an equal protein level. The co-expression of half wt and half H352F proteins leads to a dark activity comparable to ~ 1/4 of wt-only-expressing membranes (Fig. 5b). This suggested that the homo-dimerization of wt contributes to the observed 25% activity here and that the hetero-dimer of wt and H352F is non-functional, like the mutant homo-dimer. This means that *Cr*2c-Cyclop1 needs to function as dimer and that a mutation in only one half of the dimer is able to inhibit the overall activity.

**Fig. 6:**
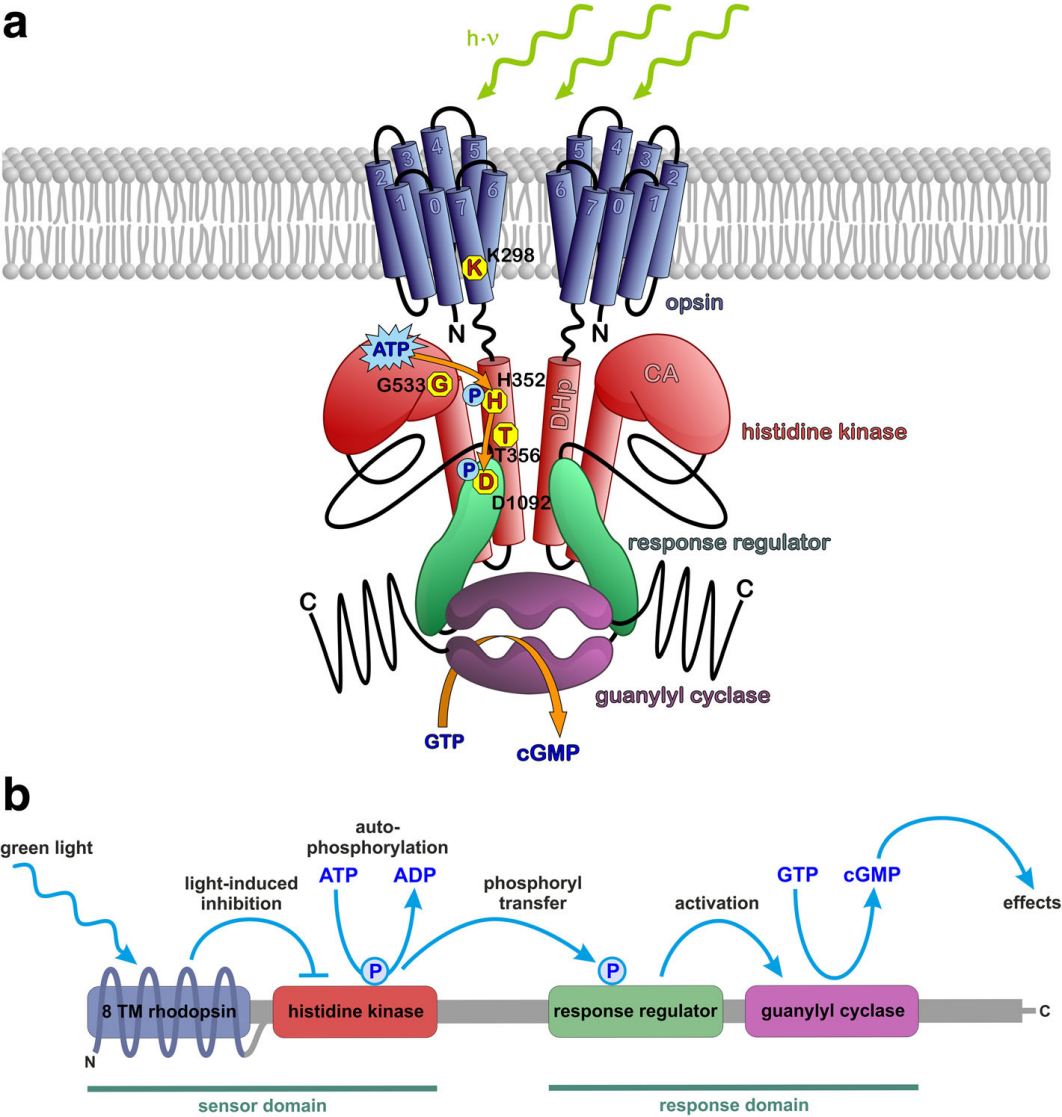
Schematic of the 2c-Cyclop working model. **a** Scheme of the *Cr*2c-Cyclop1 structure with important amino acids. Rhodopsin domain is embedded in the membrane with both termini in the cytosolic side. The key K298 residue, located in the last transmembrane helix, binds retinal covalently. Histidine kinase domain is depicted with DHp (dimerization and histidine phosphotransferase domain) and CA (catalytic and ATPase domain) in the red modules, including key residues H352, T356, and G533. Response regulator is drawn in a green module with key D1092 residue to accept phosphoryl group. Guanylyl cyclase (GC) domain is illustrated in the purple module, producing cGMP from substrate GTP. **b** A model for the cascade reaction within 2c-Cyclops. Green light is detected by the 8 TM rhodopsin, which in turn inhibits the histidine kinase. Without inhibition, the histidine kinase performs autophosphorylation using a phosphoryl group from ATP and then it transfers the phosphoryl group to the response regulator. The phosphorylated response regulator in turn activates the guanylyl cyclase to produce cGMP from GTP. The cGMP then acts as an effector molecule to trigger cellular processes

Taken the above results together, we could clarify the regulation mechanism inside *Cr*2c-Cyclop1. Both phosphorylation and phosphoryl transfer are going on in the dark between the histidine kinase and the response regulator to keep the cyclase functional. Illumination (very likely to cause a conformational change of the rhodopsin domain) will then inhibit the phosphorylation or phosphoryl transfer to slow down the cyclase activity (Fig. 6).

### Live cell imaging of *Vc*2c-Cyclop1 in *V. carteri*

**Fig. 7:**
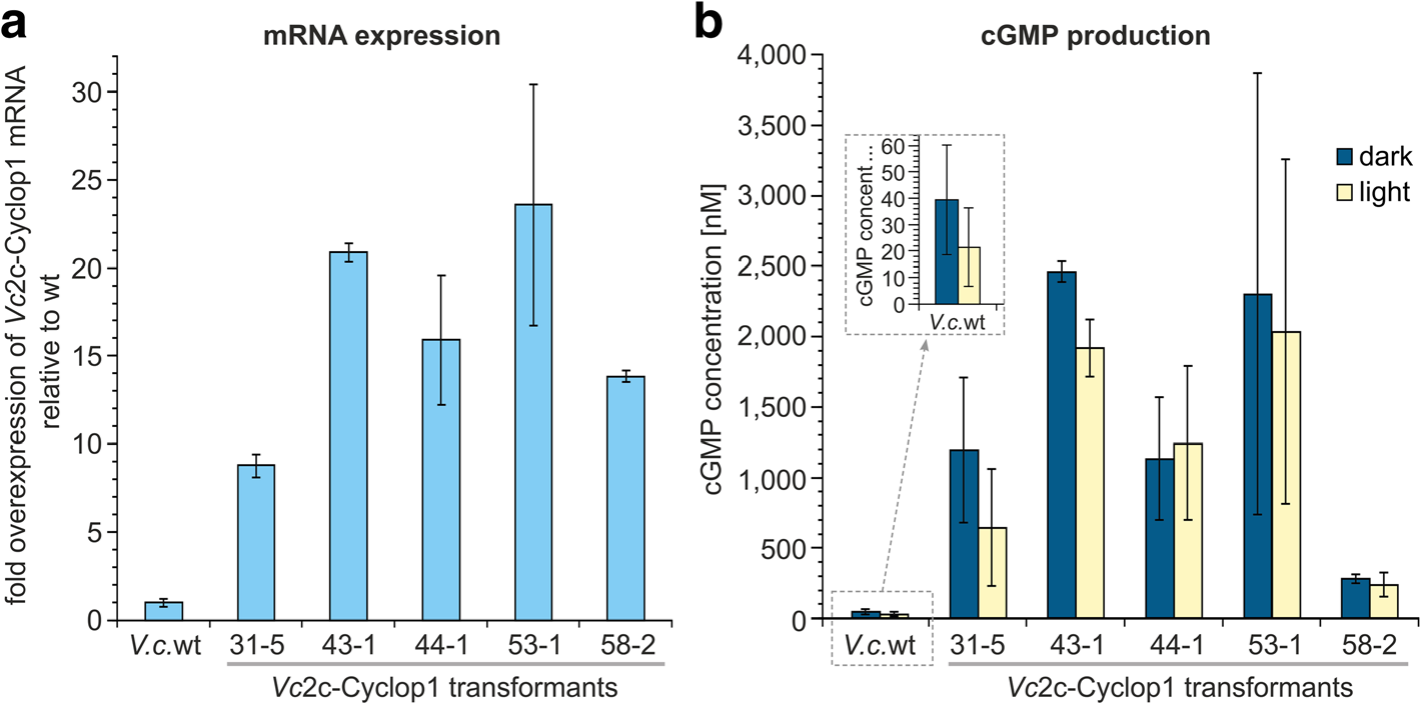
mRNA expression analysis and guanylyl cyclase activity of *Vc*2c-Cyclop1 in *V. carteri*. **a** Quantitative analysis of *Vc2c-Cyclop1* mRNA expression in wild type (wt) and in transformants that express *Vc2c-Cyclop1-YFP* under the control of the *LHCBM1* promoter. The mRNA quantification was done by quantitative real-time RT-PCR. The expression of *Vc2c-Cyclop1* in wild type was used as a reference point (=1) for calculation of the relative expression level of each transformant. The error bars represent the standard deviation of three biological replicates each. **b** Quantitative analysis of cGMP production in wild type (wt) and in transformants that express *Vc*2c-Cyclop1-YFP. The cGMP concentration serves as a measure of guanylyl cyclase activity. Wild-type and transformant *V. carteri* algae were grown under standard conditions at 28 °C in a cycle of day and night and finally analyzed during the day phase. Cell lysates were prepared both from algae samples that were transferred to the dark for 10 min (dark) and from algae samples that remained in the light during these 10 min (light). The cGMP concentration was determined in the cell lysates as described in the “Methods” section. The error bars refer to the standard deviation of three biological replicates each

For in vivo localization of the guanylyl cyclase in the source organism, we focused on *Vc*2c-Cyclop1 because we expected better localization results in *Volvox* for reasons of cell size: The largest cells in *Volvox*, the reproductive cells, are approximately 50 μm in diameter, whereas *Chlamydomonas* cells usually are only about 10 μm in diameter, which corresponds to a 125-fold difference in volume. For detectable homologous expression of *Vc*2c-Cyclop1 in *Volvox*, the complete coding sequence of *Vc*2c-Cyclop1 was fused to the YFP coding sequence. The DNA construct also contains the first two introns of the *Vc*2c-Cyclop1 gene because introns promote expression in *V. carteri* [35]. The YFP-tagged *Vc*2c-Cyclop1 was expressed under control of the *LHCBM1* promoter of *V. carteri*. In contrast to the quite weak promoter of *Vc*2c-Cyclop1, this promoter allows for significant expression of fluorescent proteins as required for cLSM localization in both cell types. For transformation, a logarithmically growing culture of a nitrate reductase-deficient strain of *V. carteri* with otherwise wild-type phenotype was grown in medium supplemented with ammonium. The culture was harvested by filtration and subjected to particle bombardment using DNA-coated gold microprojectiles as described before [36–39]. An unselectable plasmid containing the YFP-tagged *Vc*2c-Cyclop1 gene was co-transformed with plasmid pVcNR15 [35], which contains the selectable *V. carteri* nitrate reductase gene. Selection was done by growing the transformed culture in medium lacking ammonium and containing only nitrate as a nitrogen source. The obtained transformants were screened for YFP fluorescence. The expression level of *Vc*2c-Cyclop1 mRNA was analyzed for all transformants with detectable YFP fluorescence. The expression of *Vc*2c-Cyclop1 mRNA in transformants was 9- to 24-fold higher than expression of native *Vc*2c-Cyclop1 mRNA in wild-type algae (Fig. 7a).

The guanylyl cyclase enzyme activity of *Vc*2c-Cyclop1 in *V. carteri* was effectively proven by comparing the cGMP production of wild-type algae with those of transformants that overexpress *Vc*2c-Cyclop1-YFP (Fig. 7b). The cGMP production in transformants and wild-type algae roughly correlates with the determined *Vc*2c-Cyclop1 mRNA expression level (Fig. 7a, b). Light inhibition was less markedly compared to the results with 2c-Cyclop analyzed in membrane fractions of *Xenopus* oocytes (Fig. 7b), probably because permanent guanylyl cyclase overexpression resulted in cGMP accumulation over a longer period in the in vivo system. Nevertheless, the results indicate that the cGMP content is lower under light conditions.

**Fig. 8:**
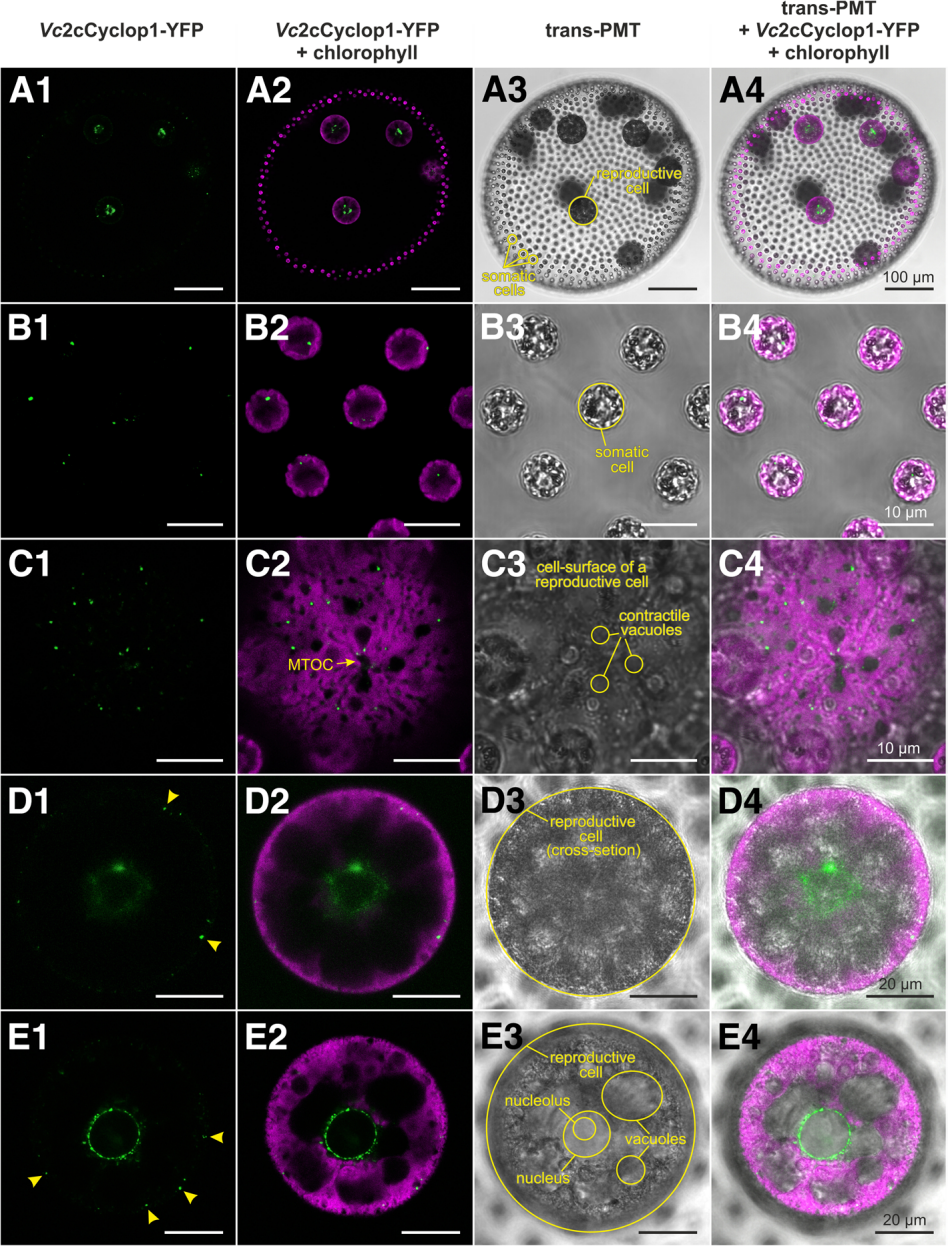
In vivo localization of *Vc*2c-Cyclop1 in *V. carteri*. **A1**–**E1** (column 1) YFP fluorescence of *Vc*2c-Cyclop1 fused to YFP (*Vc*2c-Cyclop1-YFP, green). **A2**–**E2** (column 2) Overlay of the YFP fluorescence of *Vc*2c-Cyclop1-YFP (green) and the chlorophyll fluorescence (magenta). **A3**–**E3** (column 3) Transmission-PMT image (transmitted light). **A4**–**E4** (column 4) Overlay of transmission-PMT, YFP fluorescence of *Vc*2c-Cyclop1-YFP (green) and chlorophyll fluorescence (magenta). **A1**–**A4** Overview of an entire *V. carteri* spheroid expressing *Vc*2c-Cyclop1-YFP under the control of the *LHCBM1* promoter. Larger amounts of *Vc*2c-Cyclop1-YFP are located around the nuclei of reproductive cells. Note that tiny spots of *Vc*2c-Cyclop1-YFP location can be seen only with higher magnification (see below). *V. carteri* consists of approximately 2000 small, terminally differentiated, biflagellate somatic cells at the surface and approximately 16 large reproductive cells in the interior of a transparent sphere of glycoprotein-rich extracellular matrix. **B1**–**B4** Close-up view of an optical cross section of somatic cells. Each somatic cell contains one to several tiny spots of *Vc*2c-Cyclop1-YFP location. The fluorescent spots are 0.5 to 1.0 μm in diameter (**B1**). **C1**–**C4** Close-up view of the cell surface of a reproductive cell during the growth phase. There are numerous tiny spots of *Vc*2c-Cyclop1-YFP location close to the cell surface. The fluorescent spots are 0.5 to 1.0 μm in diameter (**C1**), just like the ones observed in somatic cells. The spots of *Vc*2c-Cyclop1-YFP location never overlap with the chlorophyll fluorescence (**C2**). MTOC, microtubule organizing center. **D1**–**D4** Cross section of a reproductive cell during the growth phase. Most of *Vc*2c-Cyclop1-YFP is located close to the nucleus and appears as a diffuse cloud (**D1**, **D2**). *Vc*2c-Cyclop1-YFP also is located close to the cell surface (arrowheads in **D1**), which corresponds to the spots observed in **C1**–**C4**. **E1**–**E4** Cross section of a reproductive cell shortly before onset of embryogenesis. *Vc*2c-Cyclop1-YFP forms as a distinct structure around the nucleus. It appears that the surface of the nucleus is studded with tiny beads of *Vc*2c-Cyclop1-YFP. In addition, *Vc*2c-Cyclop1-YFP is located close to the cell surface of the cell (arrowheads in **E1**). Note that this reproductive cell is more advanced in development and thus larger than the reproductive cell in **D1**–**D4**; however, the focal plane is not as deep inside the cell as in **D1**–**D4**, which makes it possible to detect several large, non-contractile vacuoles

In vivo localization of *Vc*2c-Cyclop1 in *V. carteri* transformants was possible due to the C-terminal YFP-tag (Fig. 8). In reproductive cells, *Vc*2c-Cyclop1 localizes within a thin layer at the immediate periphery of the nucleus (Fig. 8A1–A4, D1–D4, and E1–E4). The appearance of this structure changes in a development-dependent manner (compare Fig. 8D1–D4 with E1–E4). During the growth phase of reproductive cells, the YFP signal is diffuse and patchy; it appears like a diffuse cloud (Fig. 8D1–D4). However, the signal gets clearer and sharper shortly before onset of embryogenesis (Fig. 8E1–E4). It then appears that the surface of the nucleus is studded with tiny vesicle-like structures containing *Vc*2c-Cyclop1. In addition to the structure around the nucleus, there are vesicle-like structures quite close to the surface of the reproductive cell (Fig. 8B1–B4 and C1–C4). In somatic cells, only small, vesicle-like structures of < 1 μm in diameter are detectable in the peripheral part of the cells. These structures seem to be randomly distributed (Fig. 8B1–B4).

## Discussion

*C. reinhardtii* has many opsins, some already proven and some only predicted from genome sequence data [20]. The first two “chlamyopsins” Cop1 and Cop2 were cloned as supposed opsins because of their retinal binding capacity. But they are no opsins as they show no similarity to other type I or II opsins and, most importantly, they are not even membrane proteins (Additional file 1: Figure S1A). Cop1/2 should not be called “chlamyopsin” any more. Their retinal binding capacity most probably is a result of their unusually high lysine content, resulting in unspecific retinal binding via Schiff base bonds. Cop3/4 were the first functional opsins from *Chlamydomonas*, the well-studied channelopsin-1/2. We expressed the full length of Cop5 but could not observe any functional output of this protein. The opsin domain of Cop5 was expressed previously, and a very slow photocycle of this artificial protein was observed [25]. When we expressed the Cop5 opsin domain with YFP halves fused to both termini (BiFC), we could clearly demonstrate a cytosolic N- and C-termini structure, which previously escaped attention. Cop5 (or HKR1) therefore is an opsin of type Ib. In addition, starting with Cop6, we discovered a new family of microbial opsins: two-component light-inhibited guanylyl cyclase opsins, which we abbreviate “2c-Cyclop.” Gene structures, mRNA structures, and protein domain compositions of *Cr*2c-Cyclop1 and *Vc*2c-Cyclop1 have been revealed (Fig. 1).

We established the functional mechanism of 2c-Cyclop from the green algae *C. reinhardtii* (*Cr*2c-Cyclop1) and *V. carteri* (*Vc*2c-Cyclop1) by heterologous expression in animal and homologous expression in algal cells. 2c-Cyclop belongs to a new subfamily of microbial (or type I) rhodopsins with 8 TMs and light-regulated enzymatic activity (Additional file 7: Figure S7), which we propose to classify as type Ib. It is highly likely that more 2c-Cyclop proteins will be proven to be functional, even in the same organism; although for Cop5, this is very unlikely as it lacks important conserved residues in the cyclase domain (Additional file 4: Figure S4). It cannot be excluded, however, that Cop5 becomes functional as a dimer with an as yet unknown beta subunit. The other two members of the 8 TM type Ib rhodopsins are light-activated guanylyl cyclase opsins in fungi (Cyclop) and a rhodopsin phosphodiesterase (RhoPDE), all regulating cGMP in a light-dependent manner (Additional file 7: Figure S7). The three 8 TM type Ib opsins originate from organisms, which have an eyespot and flagellar motion. These organisms are unicellular, simple multicellular, or even alternating forms between unicellular and simple multicellular ones.

With 2c-Cyclop, we demonstrate a completely new and also quite unexpected light regulation of a cGMP-generating enzyme (Fig. 6b) whose precise subcellular localization, expression timing, and function in the green algae still have to be explored.

We were also able to produce transgenic *Volvox carteri* algae that overexpress *Vc*2c-Cyclop1 (Fig. 7a). When compared to wild-type algae, these *Vc*2c-Cyclop1 transformants show a significantly increased cGMP production (Fig. 7b). These results provide proof of guanylyl cyclase activity of *Vc*2c-Cyclop1, both when expressed heterologously and in the organism of origin.

In vivo localization of *Vc*2c-Cyclop1 in *V. carteri* using transgenic algae, expressing the YFP*-*tagged *Vc*2c-Cyclop1 under the control of the *LHCBM1* promoter, demonstrated that *Vc*2c-Cyclop1 is located mainly in tiny vesicle-like structures around the nuclei of reproductive cells (Fig. 8). There are also numerous such structures with *Vc*2c-Cyclop1 close to the cell surface. In addition, each somatic cell contains one to several tiny vesicle-like structures with *Vc*2c-Cyclop1. The structures observed in reproductive cells change in a development-dependent manner (Fig. 8). The cLSM observations indicate that *Vc*2c-Cyclop1 might be localized in lipid raft-like structures within ER membranes around the nucleus and in the cortical ER.

An interesting aspect of 2c-Cyclop regulation is its ATP dependency, which suggests that this protein integrates the sensing of ATP and green light. In the zoospore of *B. emersonii*, *Be*Cyclop (originally named *Be*GC1 [11]) is necessary for its reproduction, possibly through a cGMP-dependent K^+^ channel [40]. Less is known about RhoPDE and 2c-Cyclop in their original organisms. *Salpingoeca rosetta* is not studied widely enough to get some functional hint about RhoPDE. In wild-type *V. carteri* algae, expression of *Vc*2c-Cyclop1 is quite low throughout its life cycle except for a small increase during embryogenesis. The expression does not change significantly under different stress conditions, but it is moderately upregulated in response to light stress and moderately downregulated under UV-A light and yellow light [41].

It is quite possible that expression of 2c-Cyclop is spatially localized and that its function is dependent upon developmental stage. But the difficulties with studying the physiological role of 2c-Cyclop are as follows: (1) *C. reinhardtii* and *V. carteri* have many diverse photoreceptors [20, 42] such as blue light sensing cryptochrome [43, 44], phototropin [45], and channelrhodopsins [7, 42], besides 2c-Cyclops; (2) *C. reinhardtii* and *V. carteri* also have an abundant number (~ 50) of guanylyl cyclases [42, 46]; and (3) *C. reinhardtii* and *V. carteri* have more than one 2c-Cyclop in the genome (Additional file 8: Table S1) [7, 8, 20, 21, 27, 41, 47–49]. Thus, the study of 2c-Cyclop in green algae requires more knowledge of temporal and spatial expression.

It is possible to apply 2c-Cyclop as an optogenetic tool for light-inhibited cGMP production. Especially, applying 2c-Cyclop in combination with the light-regulated PDEs, RhoPDE [18], or LAPD [50] would allow a fast decrease of cGMP level by illumination and cGMP increase in the dark.

## Conclusions

We cloned *Cr*2c-Cyclop1 from *C. reinhardtii* and *Vc*2c-Cyclop1 from *V. carteri* and characterized their functions by expression in *Xenopus* oocytes. *Cr*2c-Cyclop1 and *Vc*2c-Cyclop1 were determined to be light-inhibited and ATP-dependent guanylyl cyclases, as summarized in Fig. 6. Both opsins showed cytosolic N- and C-termini, implying an 8 transmembrane helices structure, characteristic for type Ib opsins. Guanylyl cyclase activity of *Vc*2c-Cyclop1 was also demonstrated in vivo after overexpression in *V. carteri.* Live cell imaging revealed that *Vc*2c-Cyclop1-YFP is located in a development-dependent, layer-like structure at the immediate periphery of the nucleus and intense spots in the cell periphery of *V. carteri*.

## Materials and methods

### Gene cloning, construction, and sequencing

*Cr*2c-Cyclop1 was firstly cloned from *C. reinhardtii* cDNA. Due to differences between the cloned sequence and the JGI database sequence (Additional file 2: Figure S2), the database sequence Cre11.g467678 was synthesized by GeneArt Strings DNA Fragments (Life Technologies, Thermo Fisher Scientific) with optimized restriction sites to facilitate the following cloning and mutation. Both sequences and several derivative constructs (Additional file 2: Figure S2) were inserted into pGEMHE vector for functional comparison in *Xenopus* oocytes. *Vc*2c-Cyclop1 was cloned from *V. carteri* genomic DNA and cDNA fragments and inserted into the pGEMHE vector. Mutations were introduced into the primer sequence by PCR and ligated with existing restriction site in the sequence. BiFC constructs were made by ligating the PCR-amplified opsin part to the BiFC vector [12, 18] with introduced *Kpn*I and *Xho*I restriction sites in the primer.

All constructs were confirmed by DNA sequencing. cRNAs for *Xenopus* oocyte injection were made with the AmpliCap-MaxT7 High Yield Message Maker Kit (Epicentre Biotechnologies) using plasmids linearized by *Nhe*I digestion.

### *Xenopus* oocyte membrane extraction and in vitro reaction

After in vitro transcription, 30 ng cRNA (otherwise indicated in the figure) of different constructs were injected into *Xenopus* oocytes. Injected oocytes were then incubated in ND96 buffer (96 mM NaCl, 2 mM KCl, 1 mM CaCl_2_, 1 mM MgCl_2_, 5 mM HEPES pH 7.6) at 18 °C for 3 days. Membrane extraction was according to [12] with modified solution A. The solution A used for membrane extraction in this study contained 300 mM NaCl, 75 mM Tris-Cl, 5 mM MgCl_2_, 5 mM DTT, 5% glycerol, and 1× Protease Inhibitor Cocktail (Roche); the pH was adjusted to 7.3.

The extracted membrane was finally resuspended in solution A with a ratio of 1 oocyte to 4 μl. Four microliters of extracted membrane was added to 36 μl guanylyl cyclase reaction buffer (100 mM NaCl, 75 mM Tris-Cl pH 7.3, 5 mM MgCl_2_, 5 mM DTT, 0.2 mM GTP, and 0.25 mM ATP) for reaction. The reaction was performed at 20 °C or otherwise indicated in the figure. The reaction was stopped by addition of sample diluent containing 0.1 M HCl from the cGMP assay kit in a ratio of 1/10. The cGMP concentration (nM) was measured using the DetectX High Sensitivity Direct Cyclic GMP (or cAMP) Chemiluminescent Immunoassay Kit (Arbor assays) after proper dilution.

### Fluorescence emission detection

Fluorescence emission values were used to monitor the protein amount. The fluorescence emission values were obtained by a Fluoroskan Ascent microplate fluorometer with an excitation wavelength of 485 nm and an emission wavelength of 538 nm. The protein amount was calculated using the protocol established in [12]. For the experiments comparing mutants’ activity, fluorescence emission values were always measured to ensure equal protein amounts.

### Action spectra of *Cr*2c-Cyclop1 and *Vc*2c-Cyclop1

Lights of different wavelengths ranging from UV to red (365, 422, 460, 497, 517, 541, 563, 568, 580, 600, 641, 658, and 714 nm) were used to characterize the *Cr*2c-Cyclop1 action spectra. All light intensities were adjusted to around 0.3 μW/mm^2^, which is below the *Cr*2c-Cyclop1 half-saturation light intensity. For *Vc*2c-Cyclop1, the wavelengths used were 473, 532, 556, 596, and 635 nm. The corresponding light intensities were adjusted to approximately 0.7 μW/mm^2^. A white light source using PhotoFluor II (89 North) was applied to obtain different wavelengths in combination with narrow bandwidth interference filters (Edmund Optics). Light powers were detected by a LaserCheck photo power meter (Coherent Technologies).

The cyclase activities were measured under different light illuminations. The inhibition percentage values were calculated by (dark activity − light activity)/dark activity. Inhibition efficacy of different lights was normalized to the same number of photons.

### Imaging of *Xenopus* oocytes

Fluorescence pictures of *Xenopus* oocytes were taken with a Leica DM6000 confocal microscope.

### Bioinformatics

Clustal Omega 1.2.2 and Genedoc were used for sequence alignment and file formatting. Transmembrane helices prediction was performed with TMHMM (http://www.cbs.dtu.dk/services/TMHMM/). The secondary structure prediction for Cop1 and Cop2 was illustrated based on the NPS@ (Network Protein Sequence @nalysis) web server [NPS@: Network Protein Sequence Analysis TIBS 2000 March].

### *V. carteri* strains and culture conditions

The wild-type *Volvox carteri* f. *nagariensis* strain Eve10 (female) originates from Japan and has been described earlier [51–53]. A nitrate reductase-deficient (*nit*A^−^) descendant of this strain was generated by random mutagenesis and chlorate selection as previously described [54, 55]. This non-revertible mutant strain, TNit-1013, was used as a target in transformation experiments. Cultures were grown in modified *Volvox* medium [56] with 1 mM ammonium chloride as a nitrogen source. Cultivation was at 28 °C in a cycle of 8 h dark/16 h cool fluorescent white light [57] at an average of ~ 100 μmol photons m^−2^ s^−1^ photosynthetically active radiation (PAR) in glass tubes or Fernbach flasks. The glass tubes had caps that allow for gas exchange, and the Fernbach flasks were aerated with approximately 50 cm^3^ sterile air/min.

### Primer design

Oligonucleotide primers were designed using the primer analysis software OligoCalc [58] and Primer-BLAST [59].

### Isolation of total RNA

Approximately 250 μl of concentrated, frozen algae were grinded with a mortar and a pestle, and total RNA was extracted using 1 ml of phenol-based TRI Reagent (Sigma-Aldrich, St. Louis, MO) and 300 μl trichloromethane. RNA precipitation and RNA purification were as previously described [37].

### *V. carteri* transformation vectors

Because of its enormous size, the *Vc*2c-Cyclop1 DNA construct was amplified in parts by PCR and RT-PCR. Both *V. carteri* genomic DNA and mRNA were used as templates. The *Vc*2c-Cyclop1 part of the construct contains the first two natural introns because intronless constructs are known to show low expression [35]. The *Vc*2c-Cyclop1 gene was fused to a YFP variant, also known as mVenus, which was previously codon adapted to *Chlamydomonas* [60]. A short spacer was cloned between *Vc*2c-Cyclop1 gene and YFP gene, which codes for a pentaglycine to allow for flexibility. The *Vc*2c-Cyclop1-YFP gene was brought under control of the *LHCBM1* (Vocar.0001 s0479) promoter, which is a chlorophyll a/b binding protein of light-harvesting complex II. In previous RNAseq studies, the *LHCBM1* promoter demonstrated strong and equally high expression in both cell types [61]. As a consequence, we also used the terminator sequence of *LHCBM1*. The plasmid backbone is pUC18. The complete transformation vector is shown in Additional file 9: Figure S8.

The previously constructed plasmid pVcNR15 [35], which carries a modified *V. carteri* nitrate reductase (*nit*A) gene, was used as the selectable marker gene to rescue the *nit*A mutation in strain TNit-1013.

### Coating of microprojectiles for *V. carteri* transformation

Gold microprojectiles (1.0 μm in diameter, Bio-Rad, Hercules, CA) were coated with the required plasmids as previously described [37, 38]. The DNA-coated microprojectiles were resuspended in 60 μl EtOH and kept at 4 °C for use within 3 h.

### Stable nuclear transformation of *V. carteri*

Stable nuclear transformation of *V. carteri* females was performed using a particle gun as described [62] but with several modifications according to Hallmann and Wodniok [36]. Algae of the *V. carteri* nitrate reductase-deficient strain TNit-1013 were co-bombarded with the selectable plasmid pVcNR15 and the non-selectable *Vc*2c-Cyclop1-YFP plasmid. For selection of transformants, the nitrogen source was switched from ammonium to nitrate.

### Quantitative real-time RT-PCR in *V. carteri*

Total RNA of both transformants and wild-type algae was isolated 3 h before onset of embryogenesis. The SensiFAST SYBR Hi-Rox One-Step Kit (Bioline) and a CFX96 Touch™ Real-Time PCR Detection System (Bio-Rad) were used for real-time RNA quantification. All real-time RT-PCR experiments were carried out using three biological replicates. In addition, each biological replicate was analyzed in three technical replicates. The products of all real-time RT-PCR reactions were visualized using agarose gel electrophoresis to assure amplification of a single product of the correct size. The specific primers for amplification of a fragment of *Vc*2c-Cyclop1 were 5′-CTGGACATGGACTCTGACTG and 5′-AATGCAGTGGAGCTCATCTG. These primers bind to both native *Vc*2c-Cyclop1 and transgenic *Vc*2c-Cyclop1-YFP. The gene of the eukaryotic translation elongation factor 1α2 (*eef1*) was utilized as a reference gene using the primers 5′-GACGATTGCATGCACCACTAAG and 5′-ATCAGCAGGCACACATCAGC because *eef1* shows stable expression levels at different developmental stages and after different stress treatments in *V. carteri* [63]. Reverse transcription was carried out at 45 °C for 20 min. Amplification was performed in 40 cycles of 95 °C for 5 s, 55 °C for 10 s, and 72 °C for 8 s. Melting curves were recorded to check for amplification of a single specific product. The relative expression level was calculated using the 2^−ΔCt^ method [64, 65].

### Sample preparation for cGMP concentration measurement in *V. carteri*

Synchronized *V. carteri* cultures were grown in Fernbach flasks under normal culture conditions with standard light. Approximately 3 h before onset of embryogenesis, the Fernbach flasks were either covered with aluminum foil (dark) or cultivation was continued under normal conditions (light). After 10 min, lysates from the dark were quickly prepared under extreme low-light conditions and lysates from the light were quickly prepared under light conditions. To this end, the algae were brought to the highest possible density of organisms using a 100-μm mesh nylon screen. Then, one part of water was added and the suspension was brought to a final concentration of 0.1 M HCl for stabilization of any released cGMP. Cells were immediately disrupted and lysed in a bead mill homogenizer (Precellys Evolution, Kennesaw, GA) with a fast-spinning inner rotor and a stationary outer sheath (stator). By using 20 mg of glass beads with a diameter of 0.17 mm, the homogenizer was operated in 3 cycles of 10,000 rpm for 20 s with breaks of 10 s. For inactivation of all potentially present phosphodiesterases, samples were finally brought to 95 °C for 5 min. The cGMP concentration was assayed using the DetectX High Sensitivity Direct Cyclic GMP (or cAMP) Chemiluminescent Immunoassay Kit (Arbor assays).

### cLSM analysis of YFP-tagged *Vc*2c-Cyclop1 in V*. carteri*

*V. carteri* transformants expressing YFP-tagged *Vc*2c-Cyclop1 were examined using an inverted LSM780 confocal laser scanning microscope system (Carl Zeiss GmbH, Germany) and the ZEN digital imaging software (ZEN 2011, Carl Zeiss GmbH, Germany). Excitation was performed using the 514-nm emission line of an argon ion (Ar^+^) laser. YFP was detected between 520 and 550 nm [60], and chlorophyll was detected between 650 and 700 nm. Transmission images were obtained by using a transmission-photomultiplier tube (PMT) detector.

### Data processing

All experiments described in the manuscript were at least performed three times unless otherwise mentioned. Data were analyzed with OriginPro and Microsoft Excel. The values are presented as the means, and error bars represent the standard deviation (SD). Raw data are provided in Additional file 10.

## Additional files

Additional file 1: **Figure S1.** Expression of Cop1 and Cop2 in *Xenopus* oocytes. A. The black line indicates random coils (~ 50% for both proteins). Cylinders represent α-helices (~ 30% for both), while arrows indicate β-strands (~ 20% for both). Cop1 and Cop2 have high contents of lysine residues with 16% and 18%, respectively. Accession number from JGI databases: Cop1, Cre01.g002500.t1.1; Cop2, Cre01.g002500.t1.2. B. Fluorescence emission value of soluble fraction extracts from control oocytes and oocytes expressing YFP-Cop1, Cop1-YFP, YFP-Cop2, and chop2-YFP (ChR2-YFP). Oocytes were incubated with or without additional 1 μM ATR. Dashed line indicated background emission value from control oocyte. Twenty nanograms of cRNA was injected for each construct. *n* = 3, error bars = SD. C. Fluorescence emission value of membrane extracts from control oocytes and oocytes expressing YFP-Cop1, Cop1-YFP, YFP-Cop2, and chop2-YFP (ChR2-YFP). Oocytes were incubated with or without additional 1 μM ATR. Dashed line indicated background emission value from control oocyte. Twenty nanograms of cRNA was injected for each construct. *n* = 3, error bars = SD. (PDF 66 kb)

Additional file 2: **Figure S2.** Alignment of *Cr*2c-Cylcop1 and *Vc*2c-Cyclop1. *Cr*2c-Cylcop1 (Cre11.g467678) and *Vc*2c-Cyclop1 (Vocar.0009 s0380.1) were aligned using Clustal Omega 1.2.2. Four main domains are labeled, including opsin domain, histidine kinase (comprises DHp and CA domains), response regulator, and guanylyl cyclase. Key residues are marked in red. Yellow color backgrounded sequences in the middle and C-terminus indicate the sequences deleted for *Xenopus* oocyte characterization. See Additional file 5: Figure S5. (PDF 85 kb)

Additional file 3: **Figure S3.** Alignment of opsin domains of *Cr*2c-Cyclop1, Cop5, *Vc*2c-Cyclop1, *Be*CyclOp, and *Sr*RhoPDE with ChR2, BR, and HR. Transmembrane helices are marked with green or yellow background color. The conserved lysine residue K (covalently bound to retinal via a Schiff base) is depicted in a red box in TM7. Alignment was done by Clustal Omega. Transmembrane spanning regions were labeled according to TMHMM prediction and sequence alignments. For ChR2 (or chop2), BR, and HR, transmembrane helixes are labeled based on their structures (ChR2 PDB ID: 6EID, BR PDB ID: 5AZD, HR PDB ID: 3A7K). Cop5: Cre02.g074150, *Be*CyclOp: AIC07007.1, *Sr*RhoPDE: XP_004998010.1, ChR2 (or chop2): ABO64386, Bacteriorhodopsin (BR): WP_016329665, Halorhodopsin (HR): AAA72222.1. (PDF 57 kb)

Additional file 4: **Figure S4.** Alignment of nucleotidyl cyclase domains. Alignment of GC domains of *Cr*2c-Cyclop1, *Vc*2c-Cyclop1, Cop5, *Be*Cyclop, CYG12, Cya2, and bPAC AC domain. Blue, metal binding residues; red, base recognition residues; green, ribose-orienting residue; purple, transition state-stabilizing residue. Cop5: XP_001701623.1, *Be*CyclOp: AIC07007.1, CYG12: EDP07101.1, Cya2: WP_010871597.1, bPAC: ADC33127.1. (PDF 47 kb)

Additional file 5: **Figure S5.** Comparison of different *Cr*2c-Cyclop1 constructs. A. Schematic models of *Cr*2c-Cyclop1. *Cr*2c-Cyclop1.fl is the full length sequence from JGI database. *Cr*2c-Cyclop1.s is the sequence cloned from cDNA with deletions of a short middle part and C-terminal. *Cr*2c-Cyclop1.sc is an artificial sequence with a deletion of the C-terminal. *Cr*2c-Cyclop1.sm sc is an artificial sequence with a deletion of the short middle sequence. The gray dashed lines indicate the deleted regions. Four conserved domains are labeled with different colors. Blue, opsin domain; red (His-Kinase), histidine kinase domain; green (RR), response regulator domain; purple (GC), guanylyl cyclase domain. B. Comparison of dark and light (532 nm, ~ 20 μW/mm^2^) activities of four different constructs with different lengths; activities in the dark and light came from one oocyte membrane. Approximately thirty nanograms of cRNA were injected for all constructs, 3 dpi. *n* = 3–6, error bar = SD. Reaction buffer: 75 mM Tris-Cl, 10 mM NaCl, 5 mM MgCl_2_, 0.2 mM GTP, 0.25 mM ATP, 5 mM DTT, pH 7.3. C. The fluorescence emission values were measured for four constructs individually, 12 oocytes membrane expressing individual construct was extracted and applied for each measurement. Control values were subtracted for different samples. *n* = 3, error bar = SD. D. *Cr*2c-Cyclop1.s activity under different NaCl concentrations. Other components in the reactions are as follows: 75 mM Tris-Cl, 5 mM MgCl_2_, 0.2 mM GTP, 0.25 mM ATP, 5 mM DTT, pH 7.3. *n* = 3, error bar = SD. Samples were from two batches of oocytes. Illumination condition, 532 nm, ~ 20 μW/mm^2^ light. (PDF 63 kb)

Additional file 6: **Figure S6.** Alignment of 2c-Cyclop histidine kinase and response regulator with two typical two-component system proteins. H-box and G-box of histidine kinase domain are labeled with two black boxes. For *Cr*2c-Cyclop1, key residue H352 (red) as autophosphorylation site, T356 (violet) as predicted phosphatase site, G533 (blue) as ATP binding site, D1092 (green) as phosphoryl group accepting site. Accession No.: HK853: NP_228662.1, EnvZ: WP_069357419.1, RR468: AAD35552.1, OmpR: CDZ22180.1 (PDF 75 kb)

Additional file 7: **Figure S7.** Three classes of enzyme rhodopsins. The functional domains are labeled with five different colored boxes. Blue-colored box (opsin) indicates opsin domain. Red-colored box (His-Kinase) indicates histidine kinase domain. Green-colored box (RR) indicates response regulator domain. Purple-colored box (GC) indicates guanylyl cyclase (GC) domain, specifically catalyzing GTP to cGMP in these enzyme opsins. Light orange-colored box (PDE) indicates phosphodiesterase domain. (PDF 39 kb)

Additional file 8: **Table S1.** Hypothetical, experimentally proven, and experimentally disproven opsins in *C. reinhardtii* and in *V. carteri. (PDF 24 kb)*

Additional file 9: **Figure S8.** Vector for expression of YFP-tagged *Vc*2c-Cyclop1 in *V. carteri.* The complete coding sequence of *Vc*2c-Cyclop1 was fused to the YFP coding sequence. The DNA construct also contains the first two introns of the *Vc*2c-Cyclop1 gene. The YFP-tagged *Vc*2c-Cyclop1 was expressed under control of the LHCBM1 promoter of *V. carteri*. The plasmid backbone is pUC18. (PDF 306 kb)

Additional file 10: Raw data values. (PDF 230 kb)
