# Supplementary figures and images for: Two-component cyclase opsins of green algae are ATP-dependent and light-inhibited guanylyl cyclases
Source: BMC Biol. 2018 Dec 6;16:144. doi: 10.1186/s12915-018-0613-5 (PMC6284317; doi:10.1186/s12915-018-0613-5)

Additional file 1: Figure S1

A

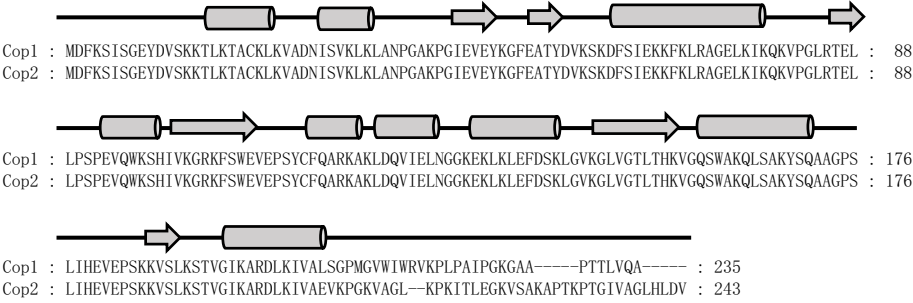

B

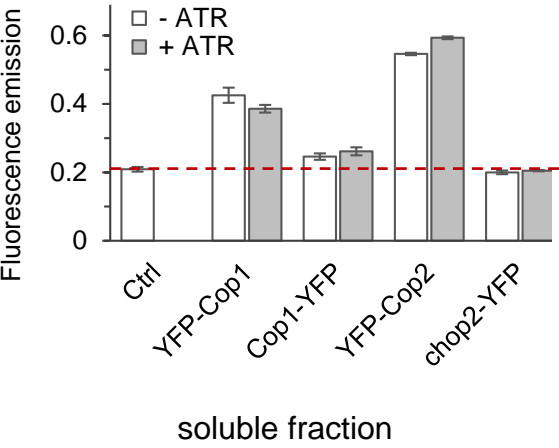

C

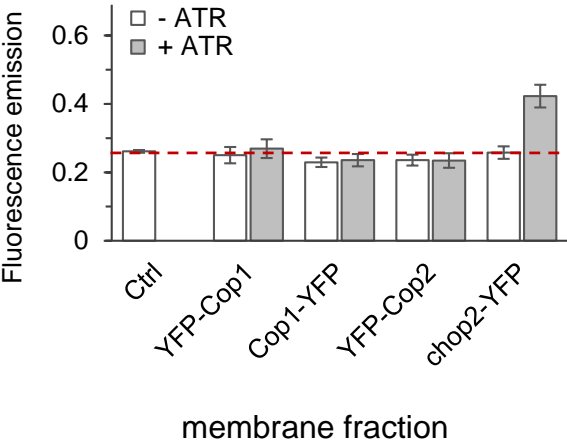

Supplement: Supplementary file 1 — Figure S1. Expression of Cop1 and Cop2 in Xenopus oocytes. A. The black line indicates random coils (~ 50% for both proteins). Cylinders represent α-helices (~ 30% for both), while arrows indicate β-strands (~ 20% for both). Cop1 and Cop2 have high contents of lysine residues with 16% and 18%, respectively. Accession number from JGI databases: Cop1, Cre01.g002500.t1.1; Cop2, Cre01.g002500.t1.2. B. Fluorescence emission value of soluble fraction extracts from control oocytes and oocytes expressing YFP-Cop1, Cop1-YFP, YFP-Cop2, and chop2-YFP (ChR2-YFP). Oocytes were incubated with or without additional 1 μM ATR. Dashed line indicated background emission value from control oocyte. Twenty nanograms of cRNA was injected for each construct. n = 3, error bars = SD. C. Fluorescence emission value of membrane extracts from control oocytes and oocytes expressing YFP-Cop1, Cop1-YFP, YFP-Cop2, and chop2-YFP (ChR2-YFP). Oocytes were incubated with or without additional 1 μM ATR. Dashed line indicated background emission value from control oocyte. Twenty nanograms of cRNA was injected for each construct. n = 3, error bars = SD. (PDF 66 kb) [file 12915_2018_613_MOESM1_ESM.pdf]

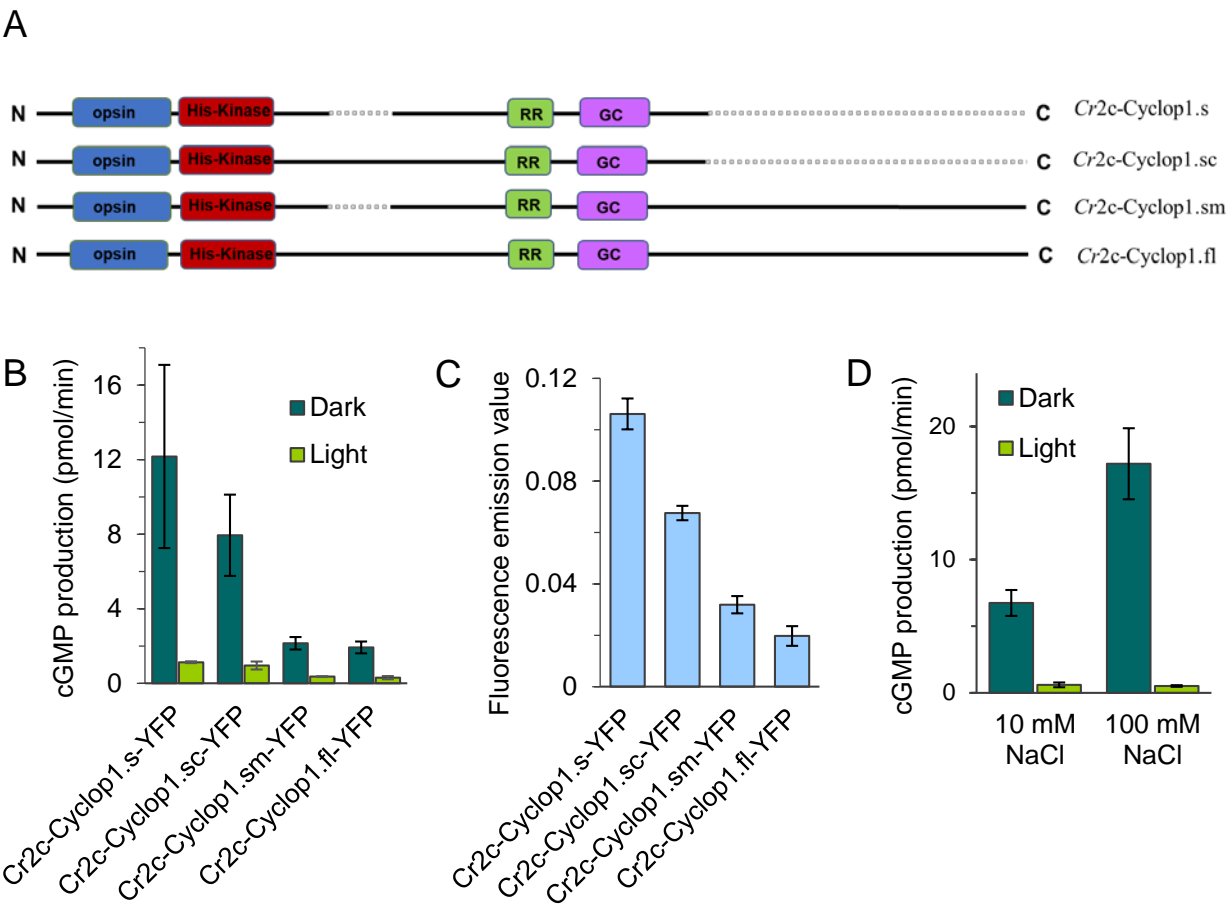

Supplement: Supplementary file 5 — Figure S5. Comparison of different Cr2c-Cyclop1 constructs. A. Schematic models of Cr2c-Cyclop1. Cr2c-Cyclop1.fl is the full length sequence from JGI database. Cr2c-Cyclop1.s is the sequence cloned from cDNA with deletions of a short middle part and C-terminal. Cr2c-Cyclop1.sc is an artificial sequence with a deletion of the C-terminal. Cr2c-Cyclop1.sm sc is an artificial sequence with a deletion of the short middle sequence. The gray dashed lines indicate the deleted regions. Four conserved domains are labeled with different colors. Blue, opsin domain; red (His-Kinase), histidine kinase domain; green (RR), response regulator domain; purple (GC), guanylyl cyclase domain. B. Comparison of dark and light (532 nm, ~ 20 μW/mm2) activities of four different constructs with different lengths; activities in the dark and light came from one oocyte membrane. Approximately thirty nanograms of cRNA were injected for all constructs, 3 dpi. n = 3–6, error bar = SD. Reaction buffer: 75 mM Tris-Cl, 10 mM NaCl, 5 mM MgCl2, 0.2 mM GTP, 0.25 mM ATP, 5 mM DTT, pH 7.3. C. The fluorescence emission values were measured for four constructs individually, 12 oocytes membrane expressing individual construct was extracted and applied for each measurement. Control values were subtracted for different samples. n = 3, error bar = SD. D. Cr2c-Cyclop1.s activity under different NaCl concentrations. Other components in the reactions are as follows: 75 mM Tris-Cl, 5 mM MgCl2, 0.2 mM GTP, 0.25 mM ATP, 5 mM DTT, pH 7.3. n = 3, error bar = SD. Samples were from two batches of oocytes. Illumination condition, 532 nm, ~ 20 μW/mm2 light. (PDF 63 kb) [file 12915_2018_613_MOESM5_ESM.pdf]

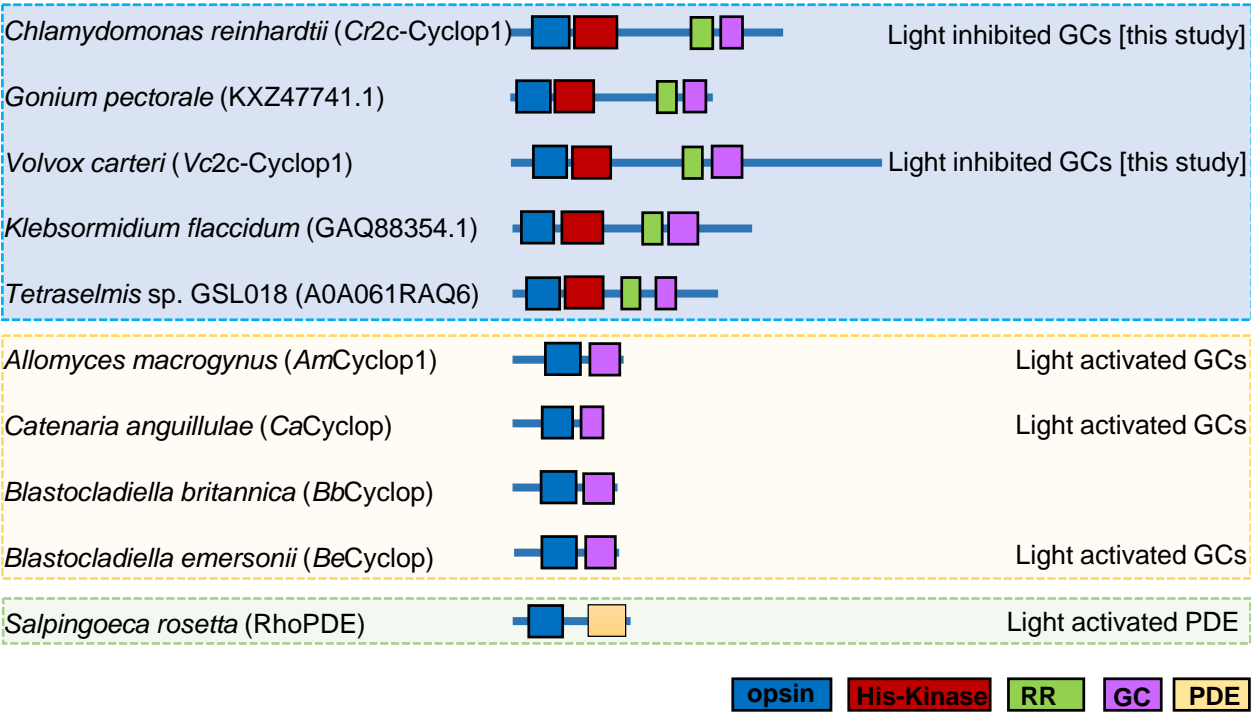

Supplement: Supplementary file 7 — Figure S7. Three classes of enzyme rhodopsins. The functional domains are labeled with five different colored boxes. Blue-colored box (opsin) indicates opsin domain. Red-colored box (His-Kinase) indicates histidine kinase domain. Green-colored box (RR) indicates response regulator domain. Purple-colored box (GC) indicates guanylyl cyclase (GC) domain, specifically catalyzing GTP to cGMP in these enzyme opsins. Light orange-colored box (PDE) indicates phosphodiesterase domain. (PDF 39 kb) [file 12915_2018_613_MOESM7_ESM.pdf]

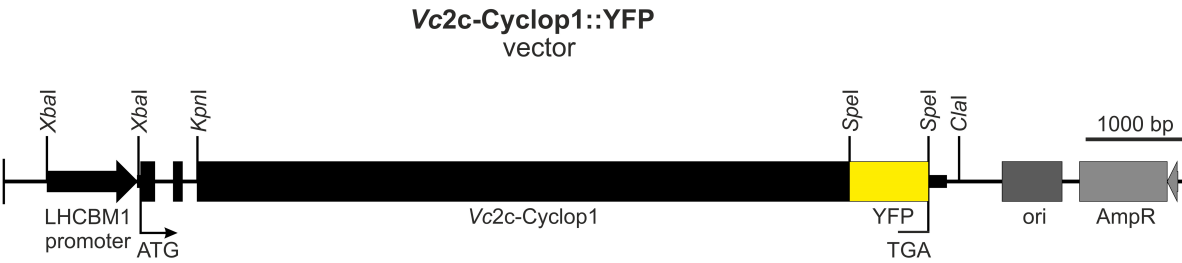

Supplement: Supplementary file 9 — Figure S8. Vector for expression of YFP-tagged Vc2c-Cyclop1 in V. carteri. The complete coding sequence of Vc2c-Cyclop1 was fused to the YFP coding sequence. The DNA construct also contains the first two introns of the Vc2c-Cyclop1 gene. The YFP-tagged Vc2c-Cyclop1 was expressed under control of the LHCBM1 promoter of V. carteri. The plasmid backbone is pUC18. (PDF 306 kb) [file 12915_2018_613_MOESM9_ESM.pdf]
