# Supplementary material for: Two-component cyclase opsins of green algae are ATP-dependent and light-inhibited guanylyl cyclases
Source: BMC Biol. 2018 Dec 6;16:144. doi: 10.1186/s12915-018-0613-5 (PMC6284317; doi:10.1186/s12915-018-0613-5)

Additional file 2: Figure S2

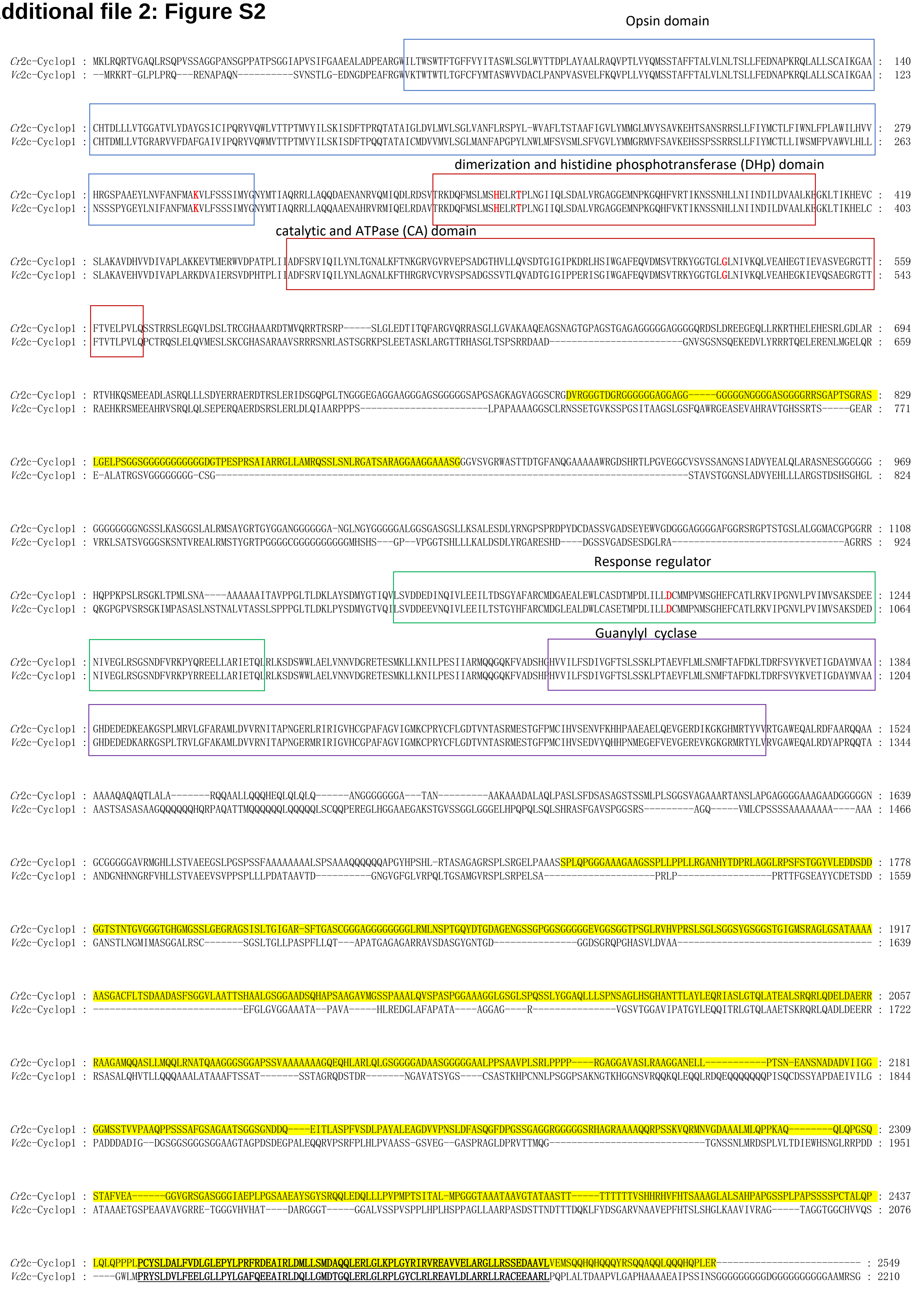

Supplement: Supplementary file 2 — Figure S2. Alignment of Cr2c-Cylcop1 and Vc2c-Cyclop1. Cr2c-Cylcop1 (Cre11.g467678) and Vc2c-Cyclop1 (Vocar.0009 s0380.1) were aligned using Clustal Omega 1.2.2. Four main domains are labeled, including opsin domain, histidine kinase (comprises DHp and CA domains), response regulator, and guanylyl cyclase. Key residues are marked in red. Yellow color backgrounded sequences in the middle and C-terminus indicate the sequences deleted for Xenopus oocyte characterization. See Additional file 5: Figure S5. (PDF 85 kb) [file 12915_2018_613_MOESM2_ESM.pdf]
