# Supplementary material for: Two-component cyclase opsins of green algae are ATP-dependent and light-inhibited guanylyl cyclases
Source: BMC Biol. 2018 Dec 6;16:144. doi: 10.1186/s12915-018-0613-5 (PMC6284317; doi:10.1186/s12915-018-0613-5)
Supplement: Supplementary file 3 — Figure S3. Alignment of opsin domains of Cr2c-Cyclop1, Cop5, Vc2c-Cyclop1, BeCyclOp, and SrRhoPDE with ChR2, BR, and HR. Transmembrane helices are marked with green or yellow background color. The conserved lysine residue K (covalently bound to retinal via a Schiff base) is depicted in a red box in TM7. Alignment was done by Clustal Omega. Transmembrane spanning regions were labeled according to TMHMM prediction and sequence alignments. For ChR2 (or chop2), BR, and HR, transmembrane helixes are labeled based on their structures (ChR2 PDB ID: 6EID, BR PDB ID: 5AZD, HR PDB ID: 3A7K). Cop5: Cre02.g074150, BeCyclOp: AIC07007.1, SrRhoPDE: XP_004998010.1, ChR2 (or chop2): ABO64386, Bacteriorhodopsin (BR): WP_016329665, Halorhodopsin (HR): AAA72222.1. (PDF 57 kb) [file 12915_2018_613_MOESM3_ESM.pdf]

Additional file 3: Figure S3

```
Cr2c-Cyclop1 : -------------------------------------------------------------------------MKLRQRTVG--AQLRSQPVSSAGG--- :  22
Cop5         : -----------------------------------------------------------------------------------------------MA--- :   2
Vc2c-Cyclop1 : ---------------------------------------------------------------------------MRKR-TG--LPLPRQ---RENA--- :  16
BeCyclop     : MKDKDNNLRGACSSCNCPEYCFSPTSTLCDDCKCSVTKHPIVEQPLSRNGSFRSSGASLLPSPSSPNVKITSTVGLRSRKSESQANVRGSMISNSNSGSR : 100
SrRhoPDE     : -------------------------------------------------------------------------MG---RKNA----ANSSMLQEASMNNY :  20
ChR2         : ---------------------------------------------MDYGGALSAVGRELLFVTNPVVVNG--S-------VLVPE--------DQC---- :  34
BR           : ----------------------------------------------------------------------------------------MRMLPELS---- :   8
HR           : -----------------------------------------------------------MTETLPPVTES--A-------VALQAEVTQRELFEFV---- :  28

                                                                    TM0
Cr2c-Cyclop1 : ---PANSGPPATPSGGIAPVSIFGAAEALADPEARGWI-------------LTWSWTFTGFFVYITASWLSGLWYTTDPLAYAALRAQVPT--LVYQMSS : 104
Cop5         : ---PTGSLPS-HLQDRIDALETNQRDTD-AQAFQKAVERRAR---------VSFSVAIAGFGVLLYLLASYLICTSNLGDPELTAQFHAQADPNAYTWPM :  88
Vc2c-Cyclop1 : ---PAQNS----------VNSTLGE-DNGDPEAFRGWV-------------KTWTWTLTGFCFYMTASWVVDACLPANPVASVELFKQVPL--LVYQMSS :  87
BeCyclop     : ---SNNSGGAGGGSGGSSS-SKGGSA---LANYQSAMSELWSWNMMLSTPSLKFLTVQFTTWIVLTTVGAIYTLFFHERQAYNRG-----WADIWYGYGA : 188
SrRhoPDE     : SMTSAASGASSSGRGKRRA-KT--------RNIAIASTKEVQW--------------QGIFMIIVWLCVMGSLIFFANPEASRRVFAKFSHLQSFYGATS :  97
ChR2         : ---------------------------------------------------------YCAGWIESR------------------GTNGAQTASNVLQWLA :  59
BR           : -------------------------------------------------------------------------------------FGEYWLVFNMLSLTI :  23
HR           : ---------------------------------------------------------LNDPL---------------------------LASSLYINIAL :  44

                    TM1                TM2                                            TM3
Cr2c-Cyclop1 : TAFFTALVLNLTSLLFEDNAPKRQLALLSCAIKGAACHTDLLLVTGG-------------------ATVLYDAYGSICIPQRYVQWLVTTPTMVYILSKI : 185
Cop5         : VAFGTAFGLNFITLLFERESAKFQLALLACYINFLAGFSDYMSWKGY-------------------APIVRDSWGQGFQLLRTVMWLLTTPAMVYLLSII : 169
Vc2c-Cyclop1 : TAFFTALVLNLTSLLFEDNAPKRQLALLSCAIKGAACHTDMLLVTGR-------------------ARVVFDAFGAIVIPQRYVQWMVTTPTMVYILSKI : 168
BeCyclop     : FGFGLGLSFAYMGFTGARNPEKKALSLCLL---GVNFISFMSYIIIM------------LR----LTPTIEGTMANPVEPARYLEWIATCPVLILLISEI : 269
SrRhoPDE     : VAFAFATGLDILAYVNAVSDEKRVLSGILAYVDGVACISYLSMA--T------------LN----LYFLVDSTQGNPVWLMRYAEWIITCPTLLYWCGLA : 179
ChR2         : AGFSILLLMFYAYQTWK--STCGWEEIYVCAIEMVKVILEFFF---------------EFKNPSMLYL----ATGHRVQWLRYAEWLLTCPVILIHLSNL : 138
BR           : AGMLAAFVFFLLARS--YVAPRYHIALYLSALIV--FIAGYHYLRIFE------SWVGAYQLQDGVYV---PTGKPFNDFYRYADWLLTVPLLLLELILV : 110
HR           : AGL-SILLFVFMTRG--LDDPRAKLIAVSTILVPVVSIASYTGLASGLTISVLEMPAGHFAEGSSVMLGGEEVDGVVTMWGRYLTWALSTPMILLALGLL : 141

                          TM4                          TM5                             TM6
Cr2c-Cyclop1 : SDFTPRQ----TATAIGLDVLMVLSGLVANF--LRSPYL-WVAFLTSTAAFIGVLYMMGLMVYSAVK-----EH---TS-------ANSRRSLLFIYMCT : 263
Cop5         : SDFSRLK----VYSVMLADVLMITFGILAFL--AYNKVMSILFYMVAWCLFAYVVHSMWSMFHASIA-----EA---RH-------DSSRVSLEVLRLFA : 248
Vc2c-Cyclop1 : SDFTPQQ----TATAICMDVVMVLSGLMANF--APGPYLNWLMFSVSMLSFVGVLYMMGRMVFSAVK-----EH---SS-------PSSRRSLLFIYMCT : 247
BeCyclop     : TQYPH-D----PYKVIVNDYALCLAGFVGAI-SAQQPWGDLAHFV-SCLCFSYVVYSLWSCFTGAID-----GE---TQ------CNVEKSGLRWIRFST : 348
SrRhoPDE     : SRADR-S----SVSDIATADALLLAG--GALSSILPSWP--AFFVFAGSFATYIYVMLHMWGMFGKAMQ---PD---FQ----PPPPLPRHALHLLRCEI : 260
ChR2         : TGLSNDY----SRRTM-GLLVSDIGTIVWG--ATSAMATGYVKVIFFCLGLCYGANTFFHAAKAYIEGYHTVPK------------GRCRQVVTGMAWLF : 219
BR           : LGLTAAR----TWNLSIKLVVASVLMLGLGYVGEV-NT-EPGPRTLWGFLSTIPFAYILYVLWVELGQAIREAK----------FGPRVLELLGATRLVL : 194
HR           : AGSN-------ATKLFTAITFDIAMCVTGLAAAL-TTSS-HLMRWFWYAISCACFLVVLYILLVEWAQDAKAA--------------GTADMFNTLKLLT : 218

                                TM7
Cr2c-Cyclop1 : LFIWNLFPLAWILHVVHR-GS---PAAEYLNVFANFMAKVLFSSSIMYGNYMTIAQRRLLAQQDAENANRV-------QMIQDLRDSV----T------- : 341
Cop5         : VGLWFTFPVIWIVVKMGLVDI---RTEEWTWCACDFLGKVMFSSSLLHGNFLTIEQRRLIAMRIVEEGNRI-------QVIQELKDLV----EQKE---- : 330
Vc2c-Cyclop1 : LLIWSMFPVAWVLHLLNS-SS---PYGEYLNIFANFMAKVLFSSSIMYGNYMTIAQRRLLAQQAAENAHRV-------RMIQELRDAV----TRKDQFMS : 332
BeCyclop     : ITTWSLFPITWFSYTSGLISF---TVAEAGFSMIDIGAKVFLTLVLVNSTVEQ-AQ-----------NQKV-------DAITAIAEELENQINNCDA--- : 423
SrRhoPDE     : VMSWSIFPLVEFLRRQGYIDF---QVGEAMNCVADYAAKVGLAMIMVNCNLEQIN------------ALRV-------QQMHSALTGMLKVMRKTNLSSS : 338
ChR2         : FVSWGMFPILFILGPEGFGVLS-VYGSTVGHTIIDLMSKNCWGLLGHYLRVLIHEHI--LIHGDIRKTTKLNIGGTEIEVETLVEDEAEAGAVP------ : 310
BR           : LMSWGFYPIAYALGTWLPGGAAQEVAIQIGYSLADLIAKPIYGLLVFAIA--RAK-S--LEEGF------------GVEAKAA----------------- : 260
HR           : VVMWLGYPIVWALGVEGIAVL-PVGVTSWGYSFLDIVAKYIFAFLLLNYL--TSNES--VVSGS------------ILDVPSASGTPADD---------- : 291
```
