# Supplementary material for: Two-component cyclase opsins of green algae are ATP-dependent and light-inhibited guanylyl cyclases
Source: BMC Biol. 2018 Dec 6;16:144. doi: 10.1186/s12915-018-0613-5 (PMC6284317; doi:10.1186/s12915-018-0613-5)
Supplement: Supplementary file 4 — Figure S4. Alignment of nucleotidyl cyclase domains. Alignment of GC domains of Cr2c-Cyclop1, Vc2c-Cyclop1, Cop5, BeCyclop, CYG12, Cya2, and bPAC AC domain. Blue, metal binding residues; red, base recognition residues; green, ribose-orienting residue; purple, transition state-stabilizing residue. Cop5: XP_001701623.1, BeCyclOp: AIC07007.1, CYG12: EDP07101.1, Cya2: WP_010871597.1, bPAC: ADC33127.1. (PDF 47 kb) [file 12915_2018_613_MOESM4_ESM.pdf]

Additional file 4: Figure S4

```
Cr2c-Cyclop1 : -------HVVILFSDIVGFTSLSSKLPTAEVFLMLSNMFTAFDKLTDRF-SVYKVETIGDAYMVAAGHDE : 1272
Vc2c-Cyclop1 : -------HVVILFSDIVGFTSLSSKLPTAEVFLMLSNMFTAFDKLTDRF-SVYKVETIGDAYMVAAGHDE : 1208
Cop5         : ----------LLEVRLVNLGDLLASVPASDLLVALASLFHDLDTLLEQH-GCYLLEGLDESHLIVSGLDN : 973
BeCyclop     : -EAKEYESVTVFFSDITNFTVISSRTSTKDMMATLNKLWLEYDAIAKRW-GVYKVETIGDAYLGVTGAPD : 511
CYG12        : -PAQEHPEATVLFSDIVGFTEIASRSSPLEVCSLLDELYQRFDAAIEEYPQLYKVETIGDAYMVVCNVTV : 537
Cya2         : --------ITILTSDLRGFTSTSEGLNPEEVVKVLNIYFGKMADVITHH-GGTIDEFMGDGILVLFGAPT : 502
bPAC         : TVEPQLVEKIIFFSDILAFSTLTEKLPVNEVVILVNRYFSICTRIISAY-GGEVTKFIGDCVMASFTKEQ : 211

Cr2c-Cyclop1 : DEDKEAKGSPLMRVLGFARAMLDVVRNITA-----PNGERLRIRIGVHCGPAFAGVIGM-KCPRYCFLGD : 1336
Vc2c-Cyclop1 : DEDKARKGSPLTRVLGFAKAMLDVVRNITA-----PNGERMRIRIGVHCGPAFAGVIGM-KCPRYCFLGD : 1272
Cop5         : V------GDQVLHALGLARSLIAAADTFAL----GGRRSKLHLAVGVHTGPAQGVLVGY-SHPLIFFTGQ : 1032
BeCyclop     : VVPDHA-----ERACNFAVDIIEMIKSFKT-----ITGESINIRIGLNSGPVTAGVLGD-LNPHWCLVGD : 570
CYG12        : PCDDHA-----DVLLEFALRMHEEASRVAS-----SLGEPVRIRVGMHSGPVVAGVVGR-KMPRFCLFGD : 596
Cya2         : SQQDDA-----LRAVACGVEMQLALREVNQQVT-GLGLQPLEMGIGINTGEVVVGNIGSEKRTKYGVVGA : 566
bPAC         : G--DAA-----IRT---SLDIISELKQLRHHVEATNPLHLLYTGIGLSYGHVIEGNMGSSLKMDHTLLGD : 271

Cr2c-Cyclop1 : TVNTASRMESTGFPMCIHVSENVFKHHPAA-EAELQEVGERDIKGKGHMRTYVV---------------- : 1389
Vc2c-Cyclop1 : TVNTASRMESTGFPMCIHVSEDVYQHHPNM-EGEFVEVGEREVKGKGRMRTYLV---------------- : 1325
Cop5         : LPAEVHMLQATCPPNCVHVSARVLESVAHS-EREHFVPAG--VMASG-ATTYLM---------------- : 1082
BeCyclop     : TVNTASRMESTSKAGHIHISESTYHFIKSK--FVTQPLDVMEVKGKGKMQTYWVLGRK------------ : 626
CYG12        : TVNTASRMESHGEAGQIHISEACYCCLRSKERFEIRERGNITVKGKGTMRTYLLSP-------------- : 652
Cya2         : QVNLTYRIESYTTGGQIFISSTT--LEAAGDRVHVNGNRTVQPKGVKDPVVIWDV--------------- : 619
bPAC         : AVNVAARLEALTRQLPYALAFTAGVKKCCQAQWTFINLGAHQVKGKQEAIEVYTVNEAQKYYDTLQITQL : 341
```
