# Supplementary material for: Two-component cyclase opsins of green algae are ATP-dependent and light-inhibited guanylyl cyclases
Source: BMC Biol. 2018 Dec 6;16:144. doi: 10.1186/s12915-018-0613-5 (PMC6284317; doi:10.1186/s12915-018-0613-5)
Supplement: Supplementary file 6 — Figure S6. Alignment of 2c-Cyclop histidine kinase and response regulator with two typical two-component system proteins. H-box and G-box of histidine kinase domain are labeled with two black boxes. For Cr2c-Cyclop1, key residue H352 (red) as autophosphorylation site, T356 (violet) as predicted phosphatase site, G533 (blue) as ATP binding site, D1092 (green) as phosphoryl group accepting site. Accession No.: HK853: NP_228662.1, EnvZ: WP_069357419.1, RR468: AAD35552.1, OmpR: CDZ22180.1 (PDF 75 kb) [file 12915_2018_613_MOESM6_ESM.pdf]

Additional file 6: Figure S6

Histidine kinase

H-box

```
Cr2c-Cyclop1    : ---MIQDLRDSVTRKDQFMSLMSHELRTPLNGIIQLSDAL :  368
Vc2c-Cyclop1    : ---MIQELRDAVTRKDQFMSLMSHELRTPLNGIIQLSDAL :  352
HK853           : ESKELERLKRIDRMKTEFIANISHELRTPLTAIKAYAETI :  276
EnvZ            : FNHMAAGVKQLADDRTLLMAGVSHDLRTPLTRIRLATEMM :  259
```

G-box

```
Cr2c-Cyclop1    : TRKYGGTGLGLNIVKQLVEAHEGTIEVASVEGRGTTFTVE : 563
Vc2c-Cyclop1    : TRKYGGTGLGLNIVKQLVEAHEGKIEVQSAEGRGTTFTVT : 547
HK853           : TYEVPGTGLGLAITKEIVELHGGRIWVESEVGKGSRFFVW : 475
EnvZ            : -RTISGTGLGLAIVQRIVDNHNGMLELGTSERGGLSIRAW : 435
```

Response regulator

```
Cr2c-Cyclop1   : PDLILLDCMMPVMSGHEFCATLRKVIPGNVLPVIMVSAKSDEENIVEGLR :  1135
Vc2-cCyclop1   : PDLILLDCMMPNMSGHEFCATLRKVIPGNVLPVIMVSAKSDEDNIVEGLR :  1070
RR468          : PDLIVLDIMMPVMDGFTVLKKLQEKEEWKRIPVIVLTAKGGEEDESLALS :  96
OmpR           : FHLMVLDLMLPGEDGLSICRRLRSQ--SNPMPIIMVTAKGEEVDRIVGLE :  96
```
