# Supplementary material for: Two-component cyclase opsins of green algae are ATP-dependent and light-inhibited guanylyl cyclases
Source: BMC Biol. 2018 Dec 6;16:144. doi: 10.1186/s12915-018-0613-5 (PMC6284317; doi:10.1186/s12915-018-0613-5)
Supplement: Supplementary file 8 — Table S1. Hypothetical, experimentally proven, and experimentally disproven opsins in C. reinhardtii and in V. carteri. (PDF 24 kb) [file 12915_2018_613_MOESM8_ESM.pdf]

Additional file 8: Table S1

Hypothetical, experimentally proven, and experimentally disproven opsins in *C. reinhardtii* and in *V. carteri.*

**Chlamyopsins**				
**Name**	**Function**	**# of TM helices**	**Gene ID**	**Opsin?**
Cop1 [20, 21]	unknown	cytosolic*	Cre01.g002500.t1.1	hypothetical type II [21]<br>exp. data: no opsin*
Cop2 [20, 21]	unknown	cytosolic*	Cre01.g002500.t1.2	as above
Cop3 [20],<br>ChR1 [7]	light-gated<br>ion channel	7 (N-term. outside)	Cre14.g611300	type Ia [7]
Cop4 [20],<br>ChR2 [8]	light-gated<br>ion channel	7 (N-term. outside)	Cre02.g085257	type Ia [8]
Cop5 [20],<br>HKR1 [27]	unknown,<br>data only on opsin	8 (N-term. inside)*	Cre02.g074150	type Ib*
Cop6 [20],<br>*Cr*2c-Cyclop1*	light-inhibited<br>guanylyl cyclase*	8 (N-term. inside)*	Cre011.g467678	type Ib*
Cop7 [20]	unknown	predicted 8	Cre01.g038050	no exp. data<br>probably type 1b
Cop8 [20]	unknown	predicted 8	Cre07.g329900	as above
Cop9 [20]	unknown	predicted 8	Cre15.g643503.t1.1	as above
Cop10 [20]	unknown	predicted 8	Cre15.g643503.t1.2	as above
Cop11 [20]	unknown	predicted 8	Cre17.g733150.t1.1	as above
Cop12 [20]	unknown	predicted 8	Cre17.g733150.t1.2	as above
**Volvoxopsins**				
**Name**	**Function**	**# of TM helices**	**Gene ID**	**Opsin?**
Vop1 [47],<br>VR1 [48]	unknown	presumably<br>cytosolic	Y11204,<br>VOLCADRAFT_82492,<br>Vocar20001251m.g,<br>Vocar.0024s0227	hypothetical type II [47]<br>no exp. data:<br>prob. no opsin
VChR1 [41, 47, 48]	light-gated<br>ion channel	7 (N-term. outside)	EU285659,<br>VOLCADRAFT_40874,<br>Vocar20011905m.g,<br>Vocar.0050s0060	type Ia [48]
VChR2 [41, 48]	light-gated<br>ion channel	7 (N-term. outside)	EU285661,<br>VOLCADRAFT_79664,<br>Vocar20014833m.g,<br>Vocar.0028s0176	type Ia [48]
VcHKR1 [41, 49]	unknown	predicted 8	XM_002954752,<br>VOLCADRAFT_95631,<br>Vocar20010375m.g,<br>Vocar.0044s0018	no exp. data<br>probably type Ib
VcHKR2 [41, 49],<br>*Vc*2c-Cyclop1*	light-inhibited<br>guanylyl cyclase*	8 (N-term. inside)*	XM_002957019,<br>VOLCADRAFT_98081,<br>Vocar20005159m.g,<br>Vocar.0009s0380	type Ib*
VcHKR3 [41, 49]	unknown	predicted 8	XM_002953903,<br>VOLCADRAFT_106206,<br>Vocar20008208m,<br>Vocar.0001s0831	no exp. data<br>probably type Ib
VcHKR4 [41, 49]	unknown	predicted 8	XM_002952553,<br>VOLCADRAFT_93268,<br>Vocar20007107m,<br>Vocar.0069s0008	as above

* Proven in this study.
