# Supplementary material for: Two-component cyclase opsins of green algae are ATP-dependent and light-inhibited guanylyl cyclases
Source: BMC Biol. 2018 Dec 6;16:144. doi: 10.1186/s12915-018-0613-5 (PMC6284317; doi:10.1186/s12915-018-0613-5)
Supplement: Supplementary file 10 — Raw data values. (PDF 230 kb) [file 12915_2018_613_MOESM10_ESM.pdf]

Additional file 10: Raw data

Raw data for Figure 3

Figure 3A

	cGMP production [pmol/min]				
	individual values (n=3)			mean values	SD
WT + ATR Dark	11.17	11.78	11.36	11.43	0.31
WT + ATR Green	0.38	0.38	0.22	0.33	0.09
WT-YFP + ATR Dark	7.49	6.94	7.49	7.31	0.31
WT-YFP + ATR Green	0.22	0.30	0.22	0.25	0.05
WT-YFP no ATR Dark	7.22	7.48	6.91	7.20	0.28
WT-YFP no ATR Green	1.60	1.61	1.67	1.63	0.04

Figure 3B

wavelength (nm)	inhibited percentage [1-L/D]				
	individual values (n=3)			mean values	SD
365	0.115	0.029	0.072	0.072	0.043
422	0.283	0.342	0.306	0.310	0.030
460	0.446	0.388	0.446	0.427	0.033
497	0.610	0.577	0.618	0.602	0.022
517	0.727	0.746	0.746	0.739	0.011
541	0.758	0.804	0.803	0.788	0.026
563	0.760	0.776	0.725	0.754	0.026
568	0.552	0.558	0.552	0.555	0.003
580	0.560	0.575	0.570	0.569	0.008
600	0.538	0.538	0.568	0.548	0.006
641	0.487	0.448	0.488	0.474	0.022
658	0.426	0.341	0.257	0.342	0.085
714	0.189	0.163	0.190	0.181	0.015

Figure 3C

Blue light intensity (µW/mm^2)	inhibited percentage [1-L/D]				
	individual values (n=3)			mean values	SD
0.02	0.09	0.05	0.13	0.09	0.041
0.09	0.22	0.28	0.42	0.31	0.104
0.26	0.32	0.28	0.40	0.34	0.062
0.80	0.35	0.36	0.52	0.41	0.095
2.39	0.72	0.64	0.70	0.69	0.040
7.17	0.85	0.85	0.88	0.86	0.019
21.50	0.88	0.84	0.86	0.86	0.022

Green light intensity (µW/mm^2)				mean values	SD
0.02	0.16	0.08	0.22	0.12	0.052
0.09	0.41	0.47	0.56	0.48	0.074
0.26	0.55	0.60	0.67	0.61	0.062
0.80	0.71	0.76	0.73	0.74	0.024
2.39	0.88	0.87	0.90	0.88	0.018
7.17	0.93	0.93	0.93	0.93	0.002
21.50	0.97	0.96	0.96	0.96	0.005

Red light intensity (µW/mm^2)				mean values	SD
0.03	0.10	0.21	0.05	0.12	0.079
0.10	0.28	0.36	0.19	0.28	0.083
0.33	0.40	0.40	0.53	0.44	0.074
0.92	0.83	0.76	0.76	0.78	0.038
2.79	0.93	0.92	0.92	0.92	0.004
5.41	0.96	0.95	0.96	0.95	0.005
18.71	0.99	0.96	0.97	0.97	0.016

Figure 3D

	cGMP production [pmol/min]					
	individual values (n=4)				mean values	SD
Dark	0.76	0.76	0.58	0.70	0.70	0.087
Green light	0.10	0.09	0.21	0.12	0.13	0.056

Figure 3E

	inhibited percentage [1-L/D]					
wavelength (nm)	individual values (n=4)				mean values	SD
473	0.09	0.03	0.24	0.29	0.16	0.120
532	0.26	0.37	0.30	0.50	0.36	0.104
556	0.48	0.51	0.40	0.40	0.45	0.054
596	0.23	0.29	0.28	0.23	0.26	0.030
635	0.11	0.14	0.22	0.11	0.15	0.050

Figure 3F

	inhibited percentage [1-L/D]					
Green light intensity (µW/mm^2)	individual values (n=4)				mean values	SD
1.27	0.38	0.54	0.45	0.47	0.46	0.064
3.62	0.57	0.63	0.64	0.54	0.59	0.048
10.51	0.68	0.81	0.82	0.72	0.76	0.068
30.65	0.82	0.75	0.79	0.79	0.79	0.026
85.19	0.85	0.87	0.91	0.84	0.87	0.032

Raw data for Figure 4

Figure 4A

Dark

time (min)	cGMP production [pmol/sample] individual values (n=3)			mean values	SD
0.5	14.08	14.76	13.80	14.21	0.49
3.5	78.80	52.40	66.00	65.73	13.20
10	235.40	245.60	221.20	234.07	12.25

Green

time (min)				mean values	SD
0.5	15.36	12.32	12.00	13.23	1.85
3.5	17.60	23.56	18.68	19.95	3.18
10	20.08	37.84	19.32	25.75	10.48

Green->Dark

time (min)				mean values	SD
0.5	15.36	12.32	12.00	13.23	1.85
3.5	17.60	23.56	18.68	19.95	3.18
4	25.96	16.56	25.04	22.52	5.18
4.5	32.80	38.76	36.32	35.96	3.00
5.5	57.12	56.68	53.68	55.83	1.87
7.5	95.20	65.36	90.24	83.60	15.99
10	138.00	181.76	155.04	158.27	22.06

Figure 4B

	cGMP production [pmol/min] dark, individual values (n=4)				mean values	SD
standard-dark	19.07	18.67	24.50	26.40	22.16	3.88
standard-light	0.61	0.37	0.41	0.77	0.54	0.19
no $MgCl_2$-dark	0	0	0	0	0	0
no $MgCl_2$-light	0	0	0	0	0	0
5 mM $CaCl_2$-dark	0.44	0.41	0.49	0.49	0.46	0.04
5 mM $CaCl_2$-light	0.20	0.24	0.41	0.13	0.24	0.12
no ATP dark	0.68	0.36	1.49		0.84	0.59
no ATP green	0.68	0.58	0.92		0.72	0.17

Figure 4C

Temperature [°C]	cGMP production [pmol/min] dark, individual values (n=3)			mean values	SD	cGMP production [pmol/min] light, individual values (n=3)			mean values	SD
10	2.10	2.26	2.26	2.20	0.09	0.07	0.56	0.08	0.24	0.28
20	26.37	26.37	22.85	25.19	2.03	1.62	1.13	1.04	1.26	0.31
30	99.68	104.48	94.88	99.68	4.80	8.70	9.34	9.02	9.02	0.32
40	28.96	28.96	25.76	27.89	1.85	12.22	15.78	10.94	12.98	2.50

Figure 4D

pH values	dark, individual values (n=3)			mean values	SD	light, individual values (n=3)			mean values	SD
6.3	7.57	6.75	5.94	6.75	0.82	0.65	0.72	0.72	0.70	0.04
7.3	26.37	26.37	22.85	25.19	2.03	1.62	1.13	1.04	1.26	0.31
8.3	13.82	14.14	11.87	13.28	1.23	1.20	2.18	2.49	1.95	0.67

Raw data for Figure 5

Figure 5A	cGMP production [pmol/min] dark, individual values (n=4)				mean values	SD	cGMP production [pmol/min] light, individual values (n=4)				mean values	SD
WT	6.82	6.28	6.14	6.40	6.41	0.293	0.36	0.03	0.31	0.08	0.20	0.165
WT-ATP	0.08	0.33	0.11	0.28	0.20	0.122	0.14	0.11	0.08	0.17	0.13	0.036
WT+AMP-PNP	0.02	0.11	0.11	0.06	0.08	0.044	0.11	0.04	0.06	0.07	0.07	0.030
K298A	6.01	5.55	4.28	5.39	5.31	0.734	5.37	6.21	5.71	5.03	5.58	0.505
K298A-ATP	0.25	0.15	0.19	0.10	0.17	0.065	0.25	0.16	0.06	0.13	0.15	0.080
K298A+AMP-PNP	0.10	0.13	0.07	0.06	0.09	0.029	0.06	0.13	0.06	0.04	0.07	0.039
G533A	0.04	0.03	0.05		0.04	0.006	0.02	0.05	0.02		0.03	0.017
D1092T	0.07	0.06	0.08	0.09	0.08	0.015	0.08	0.06	0.10	0.02	0.06	0.032

Figure 5B Dark	cGMP production [pmol/min] dark, individual values (n=3)			mean values	SD	Light	cGMP production [pmol/min] light, individual values (n=3)			mean values	SD
wt standard	4.83	4.53	4.53	4.63	0.176	wt standard	0.23	0.15	0.42	0.27	0.136
wt+H352F	0.99	0.99	1.04	1.01	0.025	wt+H352F	0.10	0.19	0.17	0.15	0.044
H352F	0.10	0.07	0.08	0.08	0.017	H352F	0.14	0.10	0.03	0.09	0.053

Figure 5C	relative fluorescence emission values individual values (n=3)			mean values	SD
wt standard	1.07	0.91	1.02	1.00	0.079
wt+H352F	0.92	0.81	1.00	0.91	0.097
H352F	0.95	1.11	1.18	1.08	0.119

Raw data for Figure 7

Figure 7A mRNA expression
fold overexpression of *Vc* 2c-Cyclop1 mRNA relative to wt

V.c. wt and *Vc* 2c-Cyclop1 transformants	individual values (n=3-5)					mean values	SD
wt	0.8	1.2	0.9			1.0	0.2
31-5	8.4	8.4	9.6			8.8	0.7
43-1	20.4	20.9	21.4			20.9	0.5
44-1	19.5	16.1	12.2			15.9	3.7
53-1	14.1	27.8	18.6	29.2	28.3	23.6	6.8
58-2	13.5	13.9	14.2			13.9	0.3

Figure 7B cGMP production
cGMP concentration [nM] - dark

V.c. wt and *Vc* 2c-Cyclop1 transformants	individual values (n=3-5)					mean values	SD
wt	16	52	51			39.5	20.8
31-5	1620	620	1330			1190.0	514.5
43-1	2540	2410	2420			2456.7	72.3
44-1	623	1390	1370			1127.6	437.3
53-1	1360	4000	900	1220	4000	2296.0	1564.4
58-2	240	290	300			276.7	32.1

cGMP concentration [nM] - light

V.c. wt and *Vc* 2c-Cyclop1 transformants	individual values (n=3-5)					mean values	SD
wt	9	17	38			21.5	14.8
31-5	1050	220	650			640.0	415.1
43-1	2150	1800	1790			1913.3	205.0
44-1	723	1180	1810			1237.5	546.0
53-1	810	3600	970	1820	2960	2032.0	1223.3
58-2	300	140	260			233.3	83.3

Raw data for Additional file 1: Figure S1

Figure S1B

soluble fraction

	Fluorescence emission individual values (n=3)			no ATR mean values	SD	Fluorescence emission individual values (n=3)			with ATR mean values	SD
Ctrl	0.21	0.20	0.22	0.21	0.007					
YFP-Cop1	0.41	0.45	0.41	0.43	0.022	0.38	0.41	0.37	0.39	0.018
Cop1-YFP	0.26	0.24	0.25	0.25	0.009	0.27	0.25	0.26	0.26	0.011
YFP-Cop2	0.55	0.55	0.54	0.55	0.004	0.59	0.61	0.59	0.59	0.012
chop2-YFP	0.20	0.19	0.20	0.20	0.005	0.20	0.21	0.20	0.21	0.004

Figure S1C

membrane fraction

	Fluorescence emission individual values (n=3)			no ATR mean values	SD	Fluorescence emission individual values (n=3)			with ATR mean values	SD
Ctrl	0.27	0.26	0.26	0.26	0.004					
YFP-Cop1	0.27	0.25	0.23	0.25	0.024	0.28	0.27	0.25	0.27	0.014
Cop1-YFP	0.22	0.24	0.22	0.23	0.014	0.27	0.22	0.22	0.24	0.027
YFP-Cop2	0.25	0.24	0.22	0.24	0.016	0.25	0.23	0.22	0.23	0.018
chop2-YFP	0.27	0.27	0.24	0.26	0.018	0.44	0.43	0.40	0.42	0.021

Additional file 5: Figure S5

Figure S5B

Dark	cGMP production (pmol/min) individual values (n=3-6)						mean values	SD
Cr2c-Cyclop1.s	12.16	7.26	17.09				12.17	4.914
Cr2c-Cyclop1.sc	6.32	4.72	10.38	10.02	8.57	7.66	7.94	2.179
Cr2c-Cyclop1.sm	1.72	1.89	2.31	2.64	2.04	2.30	2.15	0.335
Cr2c-Cyclop1.fl	1.67	1.51	2.38	2.11	2.06	1.84	1.93	0.318

Light							mean values	SD
Cr2c-Cyclop1.s	1.13	1.13	1.20	1.07	1.17	1.10	1.13	0.049
Cr2c-Cyclop1.sc	1.13	0.63	1.14	0.94	1.13	0.80	0.96	0.214
Cr2c-Cyclop1.sm	0.34	0.34	0.40	0.37	0.37	0.35	0.36	0.025
Cr2c-Cyclop1.fl	0.46	0.30	0.20	0.27	0.32	0.28	0.30	0.086

Figure S5C	Fluorescence emission value			mean values	SD
Cr2c-Cyclop1.s	0.11	0.11	0.10	0.11	0.006
Cr2c-Cyclop1.sc	0.07	0.06	0.07	0.07	0.003
Cr2c-Cyclop1.sm	0.04	0.03	0.03	0.03	0.003
Cr2c-Cyclop1.fl	0.02	0.02	0.02	0.02	0.004

Figure S5D

Dark	cGMP production (pmol/min) individual values (n=3)			mean values	SD
10 mM NaCl	6.06	7.44	5.92	6.47	0.841
100 mM NaCl	19.09	15.31	18.19	17.53	1.970

Light				mean values	SD
10 mM NaCl	0.72	0.47	0.58	0.59	0.126
100 mM NaCl	0.47	0.55	0.52	0.51	0.041
